# Monoallelic de novo *AJAP1* loss-of-function variants disrupt trans-synaptic control of neurotransmitter release

**DOI:** 10.1126/sciadv.adk5462

**Published:** 2024-07-10

**Authors:** Simon Früh, Sami Boudkkazi, Peter Koppensteiner, Vita Sereikaite, Li-Yuan Chen, Diego Fernandez-Fernandez, Pascal D. Rem, Daniel Ulrich, Jochen Schwenk, Ziyang Chen, Elodie Le Monnier, Thorsten Fritzius, Sabrina M. Innocenti, Valérie Besseyrias, Luca Trovò, Michal Stawarski, Emanuela Argilli, Elliott H. Sherr, Bregje van Bon, Erik-Jan Kamsteeg, Maria Iascone, Alba Pilotta, Maria R. Cutrì, Mahshid S. Azamian, Andrés Hernández-García, Seema R. Lalani, Jill A. Rosenfeld, Xiaonan Zhao, Tiphanie P. Vogel, Herda Ona, Daryl A. Scott, Peter Scheiffele, Kristian Strømgaard, Mehdi Tafti, Martin Gassmann, Bernd Fakler, Ryuichi Shigemoto, Bernhard Bettler

**Affiliations:** ^1^ Department of Biomedicine, Pharmazentrum, University of Basel, Klingelbergstrasse 50, 4056 Basel, Switzerland.; ^2^ Institute of Physiology II, University of Freiburg, Hermann-Herderstrasse 7, 79104 Freiburg, Germany.; ^3^ Institute of Science and Technology Austria (IST Austria), Klosterneuburg, Austria.; ^4^ Center for Biopharmaceuticals, Department of Drug Design and Pharmacology, University of Copenhagen, Universitetsparken 2, 2100 Copenhagen, Denmark.; ^5^ Department of Biomedical Sciences, Faculty of Biology and Medicine, University of Lausanne, Rue du Bugnon 7, 1005 Lausanne, Switzerland.; ^6^ Biocenter, University of Basel, Spitalstrasse 41, 4056 Basel, Switzerland.; ^7^ Department of Neurology, University of California, San Francisco, San Francisco, CA 94158, USA.; ^8^ Institute of Human Genetics and Weill Institute for Neurosciences, University of California, San Francisco, San Francisco, CA 94158, USA.; ^9^ Department of Human Genetics, Radboud University Medical Center, Nijmegen 6525, Netherlands.; ^10^ Laboratorio Genetica Medica, ASST Papa Giovanni XXIII, Bergamo, Italy.; ^11^ UO Pediatria, Spedali Civili, Brescia, Italy.; ^12^ Department of Molecular and Human Genetics, Baylor College of Medicine, Houston, TX 77030, USA.; ^13^ Baylor Genetics, Houston, TX 77021, USA.; ^14^ Division of Rheumatology, Department of Pediatrics, Baylor College of Medicine, Houston, TX 77030, USA.; ^15^ Center for Human Immunobiology, Texas Children's Hospital, Houston, TX 77030, USA.; ^16^ Department of Molecular Physiology and Biophysics, Baylor College of Medicine, Houston, TX 77030, USA.

## Abstract

Adherens junction–associated protein 1 (AJAP1) has been implicated in brain diseases; however, a pathogenic mechanism has not been identified. AJAP1 is widely expressed in neurons and binds to γ-aminobutyric acid type B receptors (GBRs), which inhibit neurotransmitter release at most synapses in the brain. Here, we show that AJAP1 is selectively expressed in dendrites and trans-synaptically recruits GBRs to presynaptic sites of neurons expressing AJAP1. We have identified several monoallelic *AJAP1* variants in individuals with epilepsy and/or neurodevelopmental disorders. Specifically, we show that the variant p.(W183C) lacks binding to GBRs, resulting in the inability to recruit them. Ultrastructural analysis revealed significantly decreased presynaptic GBR levels in *Ajap1*^−/−^ and *Ajap1*^W183C/+^ mice. Consequently, these mice exhibited reduced GBR-mediated presynaptic inhibition at excitatory and inhibitory synapses, along with impaired synaptic plasticity. Our study reveals that AJAP1 enables the postsynaptic neuron to regulate the level of presynaptic GBR-mediated inhibition, supporting the clinical relevance of loss-of-function *AJAP1* variants.

## Teaser

*AJAP1* loss-of-function variants prevent trans-synaptic recruitment of presynaptic GBRs regulating neurotransmitter release.

## INTRODUCTION

The transmembrane protein adherens junction–associated protein 1 (AJAP1), also known as Shrew1, was initially identified as a component of adherens junctions in polarized epithelial cells (*1*). Subsequent studies revealed that AJAP1 inhibits tumor cell migration (*2*) and represents a susceptibility locus for migraine (*3*). While AJAP1 has primarily been studied in the context of cancer, AJAP1 has also been hypothesized to contribute to the development of epilepsy and/or neurodevelopmental disorders (*4*–*8*). However, insufficient knowledge of AJAP1’s function in the brain has hindered assessment of its clinical relevance. Proteomic analysis of brain tissue identified AJAP1 as a direct interaction partner of γ-aminobutyric acid type B (GABA_B_) receptors (GBRs), which are the G protein–coupled receptors for the inhibitory neurotransmitter γ-aminobutyric acid (GABA) (*9*–*11*). GBRs are expressed at most excitatory and inhibitory synapses in the brain, where they inhibit neurotransmitter release and generate slow inhibitory postsynaptic potentials that decrease neuronal excitability (*11*). Despite its interaction with GBRs, AJAP1 was not described as a synaptic protein, and its impact on GBR function remains unexplored (*12*–*15*).

GBRs are heterodimeric receptors consisting of a GB1a or GB1b subunit paired with a GB2 subunit. Both GB1a and GB1b subunits originate from the same *GABBR1* gene through differential promoter usage, whereas the GB2 subunit derives from *GABBR2.* GB1a differs from GB1b at the N terminus by the presence of two sushi domains, which are common motifs involved in protein-protein interactions (*16*). GB1a/2 receptors mainly localize to presynaptic sites, while GB1b/2 receptors are more prevalent at postsynaptic sites (*17*). The extracellular domain of AJAP1 contains a tryptophan-glycine motif at amino acid positions 183 and 184, which constitutes the central element of the binding interface with the N-terminal sushi domain of GB1a (*9*). Proteomic data thus indicate that AJAP1 forms a multiprotein complex with presynaptic GB1a/2 receptors.

Using exome sequencing and chromosomal microarray analysis, we have identified five individuals with monoallelic variants or a deletion in *AJAP1*, who present with epilepsy, neurodevelopmental problems, or intellectual disability. These clinical manifestations overlap with those of patients with pathogenic loss-of-function *GABBR1* variants (*18*). In this study, we show that AJAP1 is a dendritic protein that trans-synaptically recruits GB1a/2 receptors to presynaptic sites and reduces agonist-independent constitutive receptor activity. Through biochemical and cellular assays, we found that mouse AJAP1 variants corresponding to the human variants, p.(W183C) and p.(I271Ffs*24), exhibit a loss of function, preventing them from recruiting GBRs. Ultrastructural and electrophysiological analyses of *Ajap1*^−/−^ and heterozygous *Ajap1*^W183C/+^ mice, which harbor the W183C variant in *Ajap1*, revealed a deficiency in presynaptic GBRs. This deficiency leads to uncontrolled neurotransmitter release and impairs synaptic plasticity. These data provide a mechanistic explanation for why loss of function in both *AJAP1* and *GABBR1* causes similar synaptic dysfunctions and clinical features. Our study emphasizes that knowledge about native protein complexes aids in understanding biological processes, as well as disease mechanisms.

## RESULTS

### Biochemical characterization of de novo AJAP1 variants

**Fig. 1.**
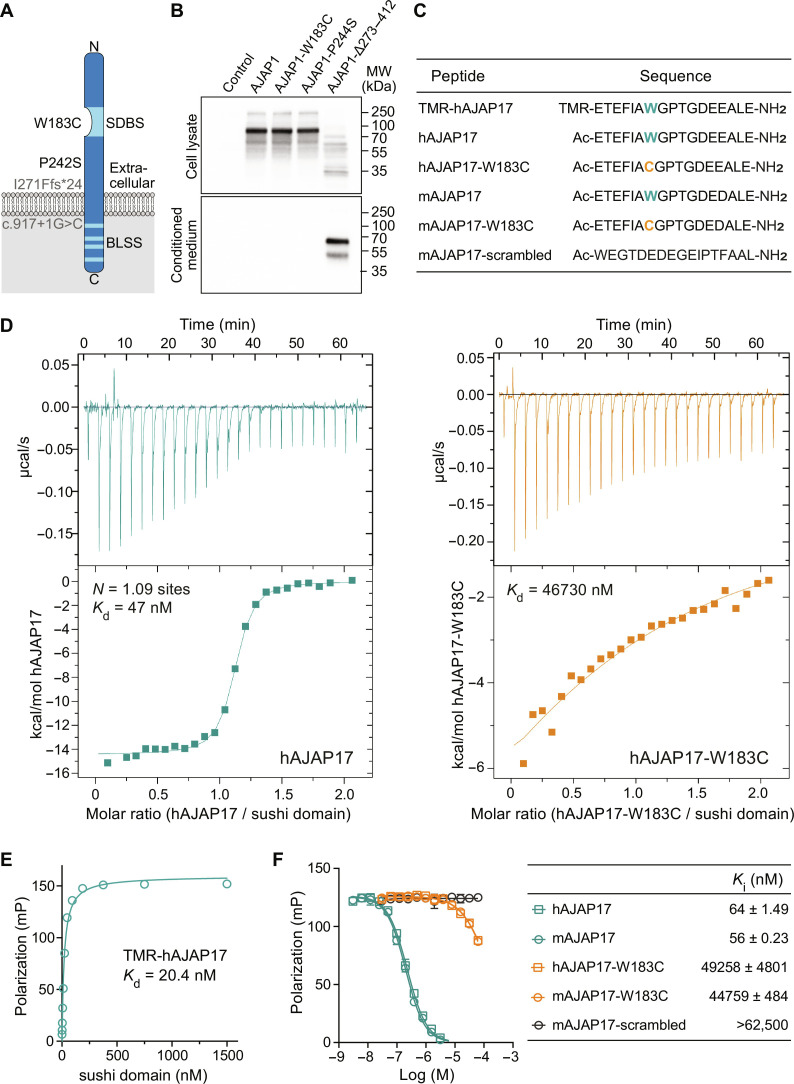
In vitro characterization of AJAP1 variants. (**A**) Scheme depicting patient AJAP1 variants. The W183C variant is located in the SDBS. The P242S variant, corresponding to mouse AJAP1-P244S, affects a residue in the extracellular domain. Hypothetical proteins are depicted in gray. The possible protein product of the p.I271Ffs*24 variant, corresponding to mouse AJAP1-Δ273–412, is hypothesized to generate a protein lacking both the transmembrane domain and the intracellular domain containing BLSS. The *AJAP1* splice variant c.917+1G>C is hypothesized to generate a protein lacking the intracellular domain after Cys306. However, both I271Ffs*24 and c.917+1G>C may also trigger nonsense-mediated decay. (**B**) Immunoblots of mouse AJAP1 variants expressed under identical conditions in HEK293T cells. Top: Cell lysates of AJAP1-, AJAP1-W183C–, and AJAP1-P244S–expressing cells. Bottom: Secreted AJAP1-Δ273–412 protein in conditioned cell culture medium. AJAP1 proteins were detected using a polyclonal anti-AJAP1 antibody (AF7970, R&D Systems). (**C**) Human (h) and mouse (m) AJAP17 and AJAP17-W183C peptides used in binding experiments. The W183C variant is highlighted in color. TMR-hAJAP17 is N-terminally labeled with the fluorophore Tamra; mAJAP17-scrambled served as a negative control in binding experiments. (**D**) Representative ITC diagrams of sushi domain protein (*9*) in solution with increasing amounts of hAJAP17 (blue) or hAJAP17-W183C (orange) peptides. Raw heat signatures (top) and integrated molar heat release (bottom) are shown. The calculated stoichiometry (*N*) and the *K*_d_ are indicated. (**E**) Saturation binding of sushi domain protein to TMR-hAJAP17 (25 nM) determined by FP analysis. (**F**) Competition of TMR-hAJAP17 (25 nM) at sushi domain protein (40 nM) by increasing concentrations of unlabeled peptides determined by FP analysis. *K*_i_ values are given in the table as means ± SEM.

Human AJAP1 is a single-pass transmembrane protein consisting of 411 amino acid residues. It is composed of an extracellular domain with the N-terminal sushi domain binding site (SDBS) (*9*), a transmembrane domain, and an intracellular domain containing basolateral sorting signals (BLSS; Fig. 1A) (*19*). Using GeneMatcher (*20*), we initially identified three individuals with epilepsy and/or neurodevelopmental phenotypes who carried heterozygous *AJAP1* variants coding for two missense variants with high Combined Annotation Dependent Depletion (CADD) scores (≥20) (*21*), and one frameshift variant: p.(W183C), p.(P242S), and p.(I271Ffs*24) (Table 1, individuals 1 to 3). More recently, we identified two additional individuals with epilepsy and/or neurodevelopmental phenotypes who carry an *AJAP1* splice variant (CADD score 35) or a deletion (Table 1, individuals 4 and 5). Overall, four of the five individuals exhibited epileptic seizures, four individuals displayed developmental phenotypes, and three individuals presented with intellectual disability.

**Table 1.** Molecular and clinical description of individuals carrying *AJAP1* variants of uncertain significance. ND, not determined; OFC, occipital frontal circumference; PDA, patent ductus arteriosus; PFO, patent foramen ovale; COPD, chronic obstructive pulmonary disease.

Individual	Individual 1	Individual 2	Individual 3	Individual 4	Individual 5
*AJAP1* variant [NM_018836.4], hg19	c.549G>T	c.724C>T	c.811del	*AJAP1* deletion chr1: 4,565,607-5,444,103	c.917+1G>C
	p.Trp183Cys	p.Pro242Ser	p.Ile271Phefs*24		
Evaluation of pathogenicity*	Pathogenic	Benign	Likely pathogenic	Likely pathogenic	Unknown
CADD score	25.6	21.4	−	−	35
Sex	F	M	M	M	F
Age	4 years	28 years	37 years	12 years 6 months	11 years
Inheritance	De novo	Nonmaternal (father not available)	De novo	Paternal (mosaic)	De novo
Other variants	−	−	De novo c.3938A>G [NM_058243.2], p.Gl n1313Arg in *BRD4*	Paternal (mosaic) deletion of exons 4–6 of *CAMTA1* (NM_015215.4; chr1:7030062-7589562)	De novo c.91C>T [NM_006937.4], p.Gln31* variant in *SUMO2*
**Developmental phenotypes**					
Global developmental delay	−	+	+	ND	ND
Motor delay	−	+	+	ND	ND
Speech delay	−	Nonverbal	Nonverbal	+	+
Ocular/visual problems	−	+	+	+	ND
Muscle tone	Normal	Hypertonia	Hypotonia	Hypotonia	Hypotonia
Scoliosis	−	+	+	ND	ND
Stereotypical / abnormal movements	−	+	+	ND	ND
Epilepsy	Febrile and afebrile seizures accompanied by postictal paresis, under Depakine treat ment long seizure-free periods and no afebrile seizures	Daily seizures, focal tonic	Simple-partial, complex-partial, and tonic-clonic, since 7 years of age	Provoked and unpro voked, generalized, under Keppra treat ment seizure-free	−
**Neuropsychiatric problems**					
Intellectual disability	ND	+	−	+	+
Autism spectrum disorder	ND	ND	+	ND	ND
Tourette syndrome	ND	ND	+	ND	ND
**Growth parameters / imaging / EEG**					
Microcephaly (OFC)	− (−1 SD)	+ (−3.9 SD at 14 years)	− (−0.07 SD)	ND	ND
Brain MRI	Normal	Multiple findings^†^	Partial agenesis of the corpus callosum, colpocephaly	Mild diffuse thinning of the corpus callosum	ND
EEG	Normal	ND	ND	ND	ND
Other phenotypes	−	Increased peripheral blood interferon score, suggestive of Aicardi- Goutières syndrome	Multiple findings^‡^	Mild pes planus	Multiple findings^§^

*On the basis of functional, predictive, and clinical data.

†Mineralization of the basal ganglia, thalami, dentate nuclei, and subcortical white matter, T2 prolongation of both the supratentorial and infratentorial white matter, supratentorial greater than infratentorial volume loss with compensatory ventricular enlargement.

‡Peters anomaly, microphthalmia, aniridia, aphakia, glaucoma, a cavernous hemangioma, dermal hypoplasia, micropenis, bilateral cryptorchidism, short stature.

§Aplasia cutis, ankyloglossia, PDA, PFO, duplex kidney, recurrent pyelonephritis, facial asymmetry, high forehead, bilateral epicanthal folds, short philtrum, micrognathia, bilateral clinodactyly of the second and third toes, hallux valgu.

The p.(W183C) missense variant affects a key residue in the SDBS (*9*) (Fig. 1A). The p.(P242S) missense variant affects a residue in the extracellular domain (Fig. 1A). The p.(I271Ffs*24) frameshift variant is located in a region of *AJAP1* in which it may trigger nonsense-mediated mRNA decay (*22*). However, the efficiency of this process varies among premature termination codons, between genes, and in different tissues (*23*–*25*). Any mRNA produced by the p.(I271Ffs*24) frameshift allele is expected to generate a truncated protein devoid of the transmembrane and intracellular domains (Fig. 1A). The *AJAP1* splice variant c.917+1G>C could lead to nonsense-mediated decay, skipping of an exon, the activation of a cryptic splice site, or the inclusion of an intron (Fig. 1A).

For experiments in human embryonic kidney (HEK) 293T cells, we introduced three variants into the mouse AJAP1 cDNA: AJAP1-W183C [p.(W183C)], AJAP1-P244S [corresponding to human p.(P242S)], and AJAP1-Δ273–412 [the orthologous variant of a possible gene product of p.(I271Ffs*24)]. AJAP1-W183C and AJAP1-P244S expressed wild-type (WT) levels of total protein (Fig. 1B) and trafficked normally to the plasma membrane (fig. S1). AJAP1-Δ273–412, lacking residues 273 to 412, was secreted into the culture medium of transfected cells (Fig. 1B).

Isothermal titration calorimetry (ITC) (*26*) demonstrated that hAJAP17, a synthetic peptide of 17 amino acid residues encoding the human AJAP1 SDBS (Fig. 1C), interacts with recombinant sushi domain protein in a 1:1 stoichiometry and a dissociation constant (*K*_d_) of 47 nM (Fig. 1D). The *K*_d_ of hAJAP17 is comparable to the *K*_d_ of 6 nM determined for full-length AJAP1 protein (*9*). In contrast, the patient variant peptide hAJAP17-W183C exhibits a *K*_d_ that is approximately 1000-fold higher. In saturation binding experiments using fluorescence polarization (FP) as a read-out, we determined a *K*_d_ of 20.4 nM for TMR-hAJAP17, a peptide labeled with the fluorophore 5,6-carboxytetramethylrhodamine (TMR; Fig. 1, C and E). In competition binding experiments, mouse and human AJAP17 peptides competed with TMR-hAJAP17 for sushi domain protein binding with similar inhibition constant (*K*_i_) values of approximately 60 nM (Fig. 1F). In contrast, mAJAP17-W183C, hAJAP17-W183C, and control mAJAP17-scrambled peptides exhibited *K*_i_ values that were more than 800-fold higher, consistent with the ITC experiments (Fig. 1F). ITC and FP assays thus support that the patient variant AJAP1-W183C no longer binds to the sushi domain protein.

### AJAP1 is expressed in the somatodendritic compartment of neurons

**Fig. 2.**
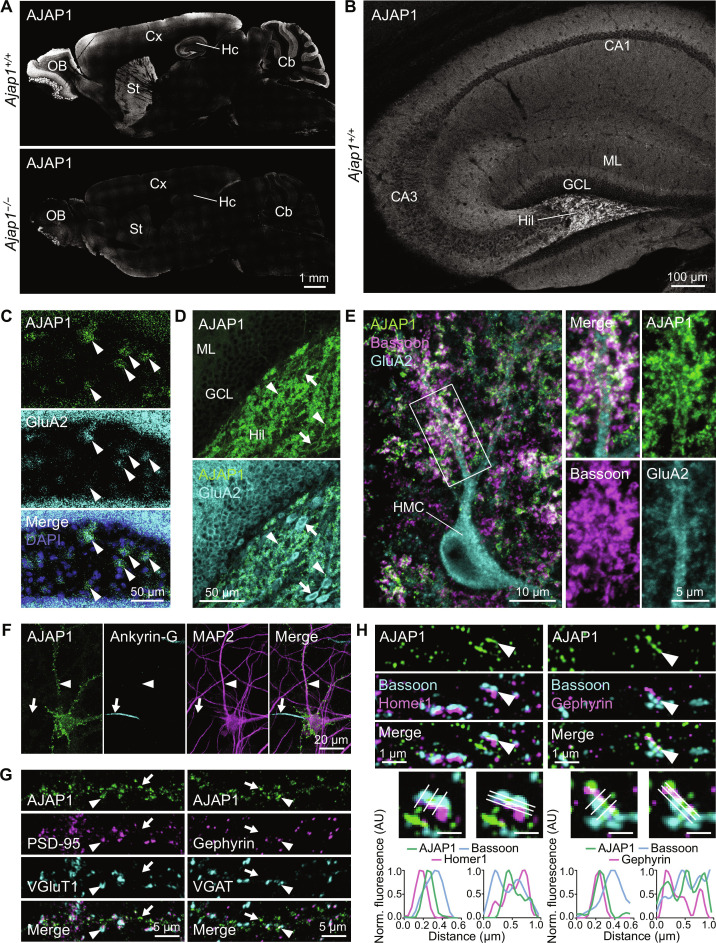
Cellular and subcellular *AJAP1* expression in mouse brain. (**A**) AJAP1 immunofluorescence in midsagittal brain section. AJAP1 is abundant in hippocampus (Hc), cerebellum (Cb), cortex (Cx), striatum (St), and olfactory bulb (OB). Antibody AF7970 specificity was controlled using sections of *Ajap1*^−/−^ mice. (**B**) Confocal image of AJAP1 immunofluorescence in midsagittal hippocampus. CA1 and CA3 subfields, molecular layer (ML), granule cell layer (GCL) and the hilus of the dentate gyrus (Hil) are labeled. (**C**) RNA-FISH for *AJAP1* and *GRIA2* (GluA2), a marker of HMCs, on coronal sections of the dentate gyrus. Colocalization of *AJAP1* and *GRIA2* transcript fluorescence in the hilus (arrowheads) suggests AJAP1 expression by HMCs. (**D**) AJAP1 immunofluorescence labeling in the dentate gyrus. AJAP1 labeling is intense in neuropil of hilus (arrowheads) and on GluA2-positive cell bodies (arrows). (**E**) AJAP1, GluA2, and Bassoon immunofluorescence at HMCs. AJAP1 localizes to synaptic structures on mossy cell dendrites adjacent to the presynaptic marker Bassoon. (**F**) Subcellular distribution of AJAP1 immunofluorescence in cultured mouse hippocampal neurons at day in vitro 21 (DIV 21). AJAP1 displays a punctate distribution in dendrites (MAP2, arrowhead) and is absent from axons (Ankyrin-G, arrow). (**G**) Dendrites of cultured hippocampal neurons immunolabeled for AJAP1 and markers for glutamatergic (PSD-95, VGluT1) and GABAergic (Gephyrin, VGAT) synapses. Arrowheads indicate colocalization, arrows denote absence of colocalization. (**H**) Structured illumination microscopy (SIM) imaging of AJAP1 in DIV 21 cultured hippocampal neurons with colabeling of the presynaptic marker Bassoon and the postsynaptic markers Homer1 and Gephyrin. AJAP1 shows a punctate distribution in proximal dendrites and partly colocalizes with synaptic markers. In selected synapses (arrowheads), AJAP1 labeling is found between pre- and postsynaptic markers, consistent with localization at the postsynaptic membrane. Graphs represent perpendicular and parallel mean fluorescence intensity profiles along the three lines of selected synapses. Bar graphs in enlarged areas, 500 nm.

We used RNA–fluorescence in situ hybridization (FISH) and immunohistochemistry to study *Ajap1* transcript and protein expression in the mouse brain. AJAP1 is widely expressed and exhibits prominent immunolabeling in various brain regions, including the hippocampus, cerebellum, cortex, striatum, and olfactory bulb (Fig. 2A). In the hippocampus, the hilus of the dentate gyrus exhibited the strongest labeling (Fig. 2B). FISH analysis revealed that AJAP1 expression in the hilus overlapped extensively with GluA2 expression, a marker of hilar mossy cells (HMCs) (*27*) (Fig. 2C). These findings align with cell type–specific RNA sequencing data that identified *Ajap1* transcripts as a marker for HMCs (*28*). AJAP1 immunolabeling was present in the somatodendritic compartment and at synapses along the dendrites of HMCs (Fig. 2, D and E). In the cerebellum, FISH revealed *Ajap1* expression in Purkinje cells (fig. S2A). Immunolabeling revealed that AJAP1 localizes to the dendrites of Purkinje cells (fig. S2B). In cultured hippocampal neurons, AJAP1 immunolabeling was similarly observed in dendrites positive for MAP2, whereas no labeling was detected in axons identified by Ankyrin-G, a marker of the axon initial segment (*29*) (Fig. 2F).

AJAP1 colocalized with postsynaptic density–95 (PSD-95) and gephyrin, which are postsynaptic markers for glutamatergic and GABAergic synapses, respectively (Fig. 2G). Structured illumination microscopy of proximal dendrites revealed the localization of AJAP1 at synapses between the presynaptic protein bassoon and the postsynaptic proteins homer1 or gephyrin (Fig. 2H). This observation is consistent with a localization of AJAP1 in the postsynaptic membrane, with the extracellular domain of AJAP1 reaching into the synaptic cleft.

Quantitative proteomic analysis of affinity purifications with several AJAP1-specific antibodies revealed an effective copurification of GB1, GB2, KCTD8, KCTD12, and KCTD16 (*9*). Likely because of constraints imposed by the antibodies used, no other proteins were copurified. Therefore, we generated a knock-in mouse with a C-terminal hemagglutinin (HA) tag in AJAP1 (*Ajap1*^HA/HA^). High-resolution mass spectrometry (MS) analysis of native AJAP1-HA complexes, isolated using two independent anti-HA antibodies, not only validated the copurification of the previously mentioned proteins but also revealed gephyrin as an interacting partner of AJAP1 (fig. S3, A and B). Gephyrin is a scaffolding protein primarily known for clustering postsynaptic glycine and GABA_A_ receptors (*30*). We corroborated the interaction of AJAP1 with gephyrin in coimmunoprecipitation experiments with transfected HEK293T cells expressing gephyrin along with either AJAP1 or AJAP1-Δ273–412 (fig. S3B). The latter is a secreted protein lacking the transmembrane and cytoplasmic domains. Anti-AJAP1 antibodies coimmunoprecipitated gephyrin with AJAP1 but not with AJAP1-Δ273–412, supporting that the intracellular domain of AJAP1 binds to gephyrin (fig. S3B). These findings provide additional support for a postsynaptic localization of AJAP1 at GABAergic synapses.

### Cytoplasmic sorting signals target AJAP1 to the dendrites

**Fig. 3.**
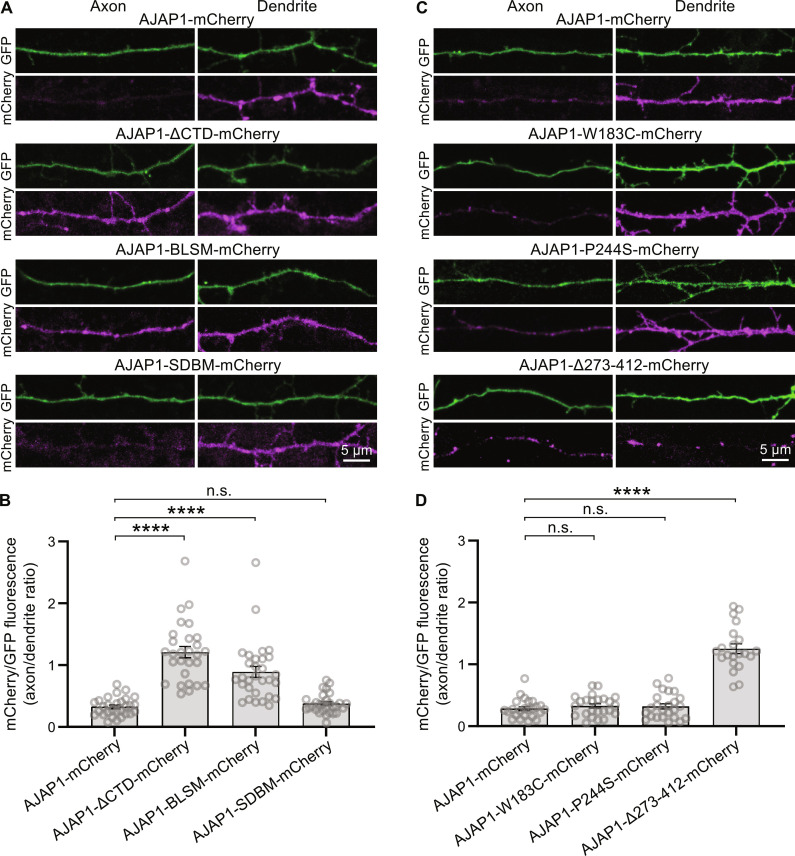
Intracellular sorting signals target AJAP1 to the dendrites. (**A**) Axonal versus dendritic sorting of AJAP1-mCherry fusion constructs expressed in cultured hippocampal neurons. AJAP1-mCherry constructs were coexpressed with the volume marker GFP at DIV 7 and fluorescence intensities quantified after 4 days. AJAP1-mCherry and AJAP1-SDBM-mCherry exhibit a dendritic localization. AJAP1-ΔCTD-mCherry and AJAP1-BLSM-mCherry lacking BLSS are distributed to axons and dendrites. (**B**) Ratios of mCherry fluorescence normalized to GFP between axons and dendrites for constructs shown in (A). ****P < 0.0001, n.s., *P* > 0.05, Kruskal-Wallis and Dunn’s multiple comparisons test, *n* = 28 to 30 cells per condition. (**C**) Subcellular sorting of AJAP1 variants expressed in cultured hippocampal neurons. AJAP1-W183C-mCherry and AJAP1-P244S-mCherry exhibit a dendritic localization. AJAP1-∆273–412-mCherry lacking BLSS is distributed to axons and dendrites. (**D**) Ratios of mCherry fluorescence normalized to GFP between axons and dendrites for constructs shown in (C). n.s., *P* > 0.05, ****P < 0.0001, Kruskal-Wallis test and Dunn’s multiple comparisons test, *n* = 21 to 24 cells per condition.

Neurons and polarized epithelial cells share common motifs for sorting membrane proteins to the somatodendritic and basolateral compartments, respectively (*31*). BLSS direct AJAP1 to adherens junctions in polarized epithelial cells (*19*). Consistent with this, our experiments demonstrated that AJAP1-mCherry localizes to the dendrites of transfected hippocampal neurons (Fig. 3, A to D). Deletion of the cytoplasmic domain in AJAP1-ΔCTD-mCherry or mutation of the BLSS in AJAP1-BLSM-mCherry resulted in random distribution of AJAP1 into both axons and dendrites in transfected hippocampal neurons (Fig. 3, A and B). Mutation of the SDBS in AJAP1-SDBM-mCherry did not disrupt the dendritic localization of AJAP1 (Fig. 3, A and B). The variants AJAP1-W183C-mCherry and AJAP1-P244S-mCherry exhibited normal dendritic distribution (Fig. 3, C and D). In contrast, the secreted variant AJAP1-Δ273–412-mCherry, lacking BLSS, showed equal trafficking to both axons and dendrites (Fig. 3, C and D). These findings provide evidence that the cytoplasmic BLSS motifs, identified in previous studies (*19*), are essential for the trafficking of AJAP1 to dendrites.

### AJAP1-W183C and AJAP1-Δ273–412 fail to allosterically regulate GBRs in trans

**Fig. 4.**
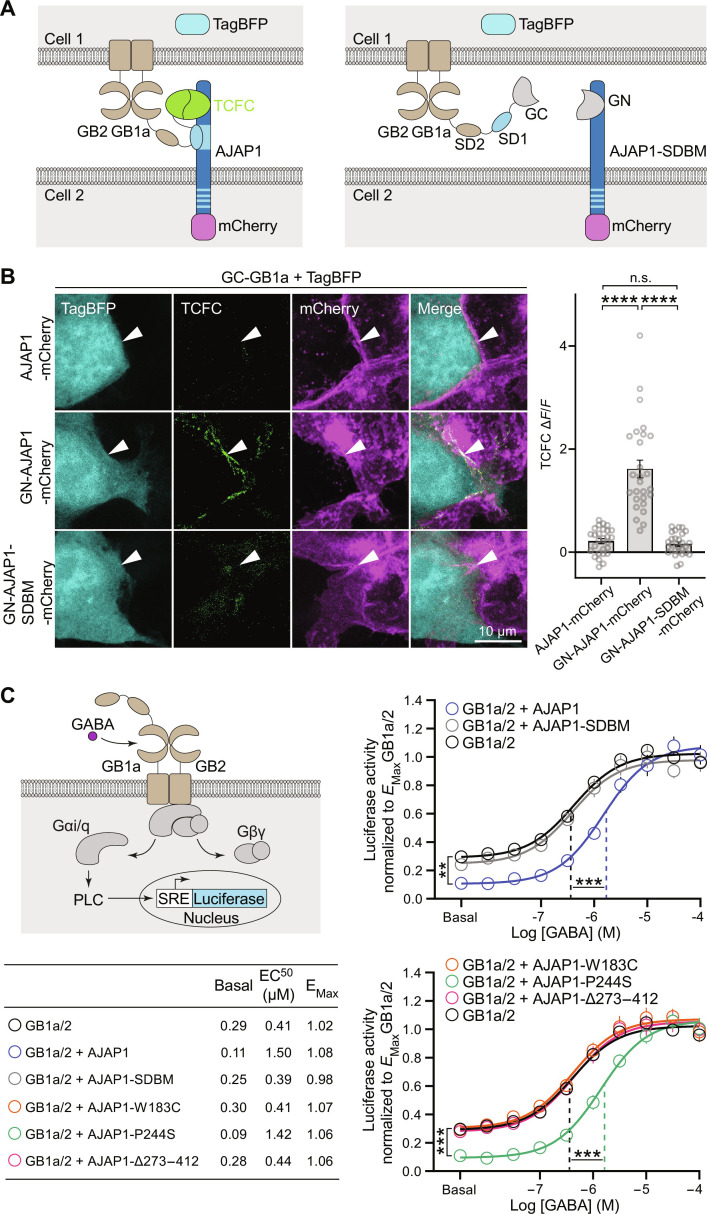
Allosteric modulation of GB1a/2 receptors by AJAP1 variants in trans. (**A**) Scheme of transcellular fluorescence complementation (TCFC) between GN-AJAP1-mCherry and GC-GB1a expressed in separate pools of transfected HEK293T cells. Binding of the N-terminal sushi domain 1 (SD1) to the SDBS of AJAP1 trans-cellularly reconstitutes the superfolder GFP fragments GFP1–10 (GN) and GFP11 (GC). AJAP1-SDBM with an inactivated SDBS does not lead to TCFC. TagBFP and mCherry identify GB1a/2 and AJAP1 expressing cells, respectively. (**B**) GN-AJAP1-mCherry but not GN-AJAP1-SDBM-mCherry or AJAP1-mCherry lead to TCFC with GC-GB1a. Arrowheads point at interfaces of AJAP1 and GB1a/2 expressing cells. For quantification of TCFC, the GFP fluorescence at cell interfaces was normalized to the background fluorescence (ΔF/F). n.s., *P* > 0.05, ****P < 0.0001, Kruskal-Wallis test, Dunn’s multiple comparisons test, *n* = 27 cells per condition. (**C**) AJAP1 is a NAM of GB1a/2 receptors in trans. Left: Assay monitoring phospholipase C (PLC)–dependent firefly luciferase expression under control of the serum response element (SRE). GB1a/2 receptors are coupled to PLC through the chimeric G protein subunit Gα_i/q_. Right: GABA-response curves of HEK293T cells expressing GB1a/2 receptors in the presence of cells with and without AJAP1 constructs. GABA-response curves of cells expressing GB1a/2 receptors exposed to cells expressing AJAP1 exhibited significantly reduced constitutive and agonist-induced receptor activity but no change in maximal efficacy (*E*_Max_, *P* = 0.196, Kruskal-Wallis test and Dunn’s multiple comparisons test). AJAP1-SDBM and the variants AJAP1-W183C and AJAP1-∆273–412 but not AJAP1-P244S failed to allosterically regulate GB1a/2 receptor activity in trans (versus GB1a/2). **P < 0.01, ***P < 0.001; one-way analysis of variance (ANOVA) and Tukey’s multiple comparisons test (constitutive activity), Kruskal-Wallis and Dunn’s multiple comparisons test (agonist-induced, EC_50_); *n* = 10 cell culture preparations per condition.

Our data suggest a trans-synaptic interaction between presynaptic GB1a and postsynaptic AJAP1. We therefore analyzed whether AJAP1 and GB1a are able to interact trans-cellularly in transfected HEK293T cells. We fused the extracellular domains of AJAP1 and GB1a with complementary GFP1–10 (GN) and GFP11 (GC) fragments of split superfolder green fluorescent protein (GFP) (*32*) (Fig. 4A). Coculturing cells expressing GN-AJAP1 with cells expressing GC-GB1a, along with GB2, resulted in trans-cellular fluorophore reconstitution (Fig. 4, A and B). As a control, GN-AJAP1-SDBM, lacking the ability to bind GB1a, did not reconstitute fluorescence with GC-GB1a.

Next, we examined the functional impact of trans-cellular interaction between AJAP1 and GB1a/2 receptors in transfected HEK293T cells. We used an accumulation assay that couples GB1a/2 receptors to a serum-responsive element-luciferase reporter via chimeric Gα_qi_ (*33*) (Fig. 4C). Cells expressing GB1a/2 receptors exposed to cells expressing AJAP1 displayed significantly reduced constitutive and agonist-induced receptor activity [increased median effective concentration (EC_50_)], while maximal efficacy (*E*_Max_) remained unchanged (Fig. 4C). These findings indicate that AJAP1 acts as a trans-cellular negative allosteric modulator (NAM) with negative intrinsic efficacy at GB1a/2 receptors. In contrast, AJAP1-SDBM, which does not bind GB1a, had no significant effect on constitutive activity or EC_50_ (Fig. 4C). The variant AJAP1-P244S retained NAM activity at GB1a/2 receptors, while AJAP1-W183C and AJAP1-Δ273–412 lacked NAM activity. Hence, NAM activity of AJAP1 in trans relies on its binding to sushi domain and its membrane anchoring.

### AJAP1-W183C and AJAP1-Δ273–412 fail to recruit neuronal GBRs in trans

**Fig. 5.**
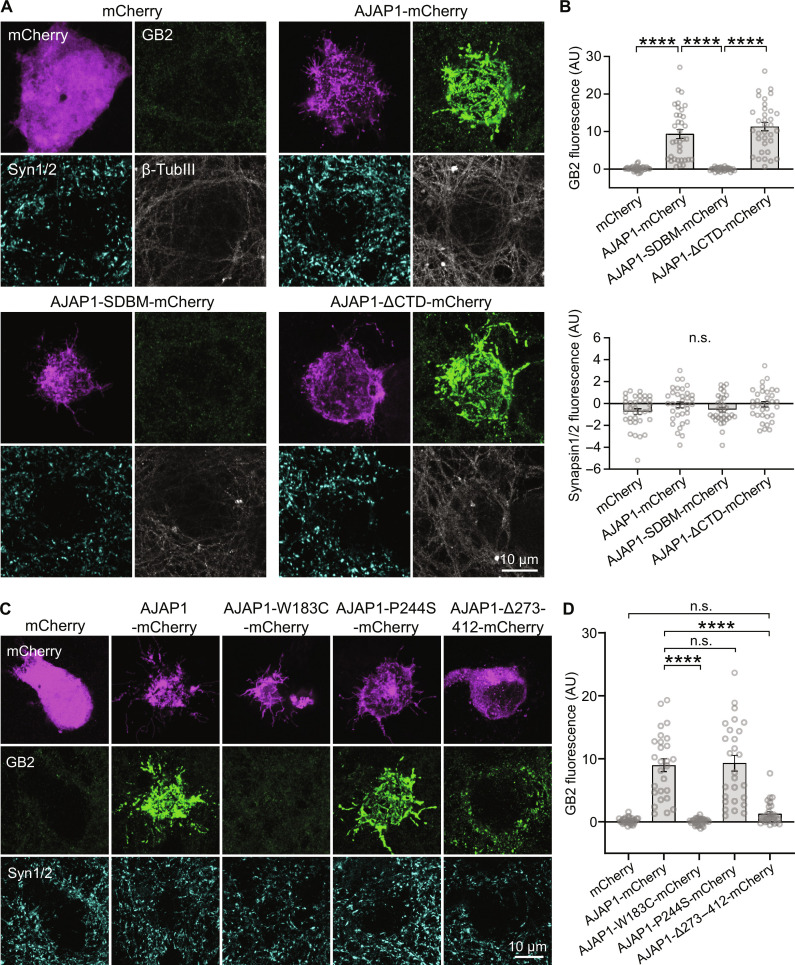
Recruitment of neuronal GBRs by AJAP1 variants in trans. (**A**) Transcellular recruitment of neuronal GBRs to HEK293T cells expressing AJAP1 constructs. HEK293T cells expressing AJAP1-mCherry, AJAP1-SDBM-mCherry or AJAP1-ΔCTD-mCherry were cocultured for 36 hours with hippocampal neurons (DIV12). Immunolabeling for GB2 shows a strong transcellular recruitment of GBRs to the soma of HEK293T cells expressing AJAP1-mCherry and AJAP1-ΔCTD-mCherry but not AJAP1-SDBM-mCherry. AJAP1-expressing cells did not recruit Synapsin1/2. β-TubulinIII was used as a marker for neurites. (**B**) GB2 and Synapsin1/2 immunofluorescence at the soma of HEK293T cells expressing AJAP1 constructs. The background fluorescence in areas devoid of transfected HEK293T cells was subtracted. n.s., *P* > 0.05, ****P < 0.0001, Welch’s ANOVA, Dunnett’s T3 multiple comparisons test (GB2), Kruskal-Wallis test and Dunn’s multiple comparisons test (Synapsin1/2), *n* = 33 to 35 cells per condition. (**C**) Transcellular recruitment of neuronal GBRs to HEK293T cells expressing AJAP1-mCherry and the variants AJAP1-W183C-mCherry, AJAP1-P244S-mCherry, and AJAP1-Δ273–412-mCherry. (**D**) GB2 immunofluorescence at the soma of HEK293T cells expressing AJAP1 variants. n.s., *P* > 0.05, ****P < 0.0001, Kruskal-Wallis test and Dunn’s multiple comparisons test, *n* = 27 to 28 cells per condition.

In a coculture assay involving cultured hippocampal neurons and HEK293T cells expressing different AJAP1 constructs, AJAP1-mCherry efficiently recruited GBRs, as identified by GB2 immunostaining, in trans (Fig. 5, A and B). However, this recruitment did not occur in the absence of AJAP1 (mCherry alone) or when AJAP1-SDBM-mCherry was present in HEK293T cells. AJAP1-ΔCTD-mCherry, which lacks the cytoplasmic domain, was as effective as AJAP1-mCherry in recruiting GBRs. Notably, the expression of AJAP1-mCherry in HEK293T cells did not induce Synapsin1/2 accumulation in contacting neurites, suggesting that AJAP1 lacks synaptogenic activity (Fig. 5, A and B). The variants AJAP1-W183C-mCherry and AJAP1-Δ273–412-mCherry failed to recruit GBR to transfected HEK293T cells (Fig. 5, C and D). The secreted AJAP1-Δ273–412-mCherry protein induced clustering of GBRs near the soma of HEK293T cells, where the concentration of the secreted protein is high. AJAP1-P244S-mCherry did not significantly differ from AJAP1-mCherry in its ability to recruit GBRs to HEK293T cells.

### Loss of GBRs in *Ajap1*^−/−^ and *Ajap1*^W183C/+^ mice impairs presynaptic inhibition

**Fig. 6.**
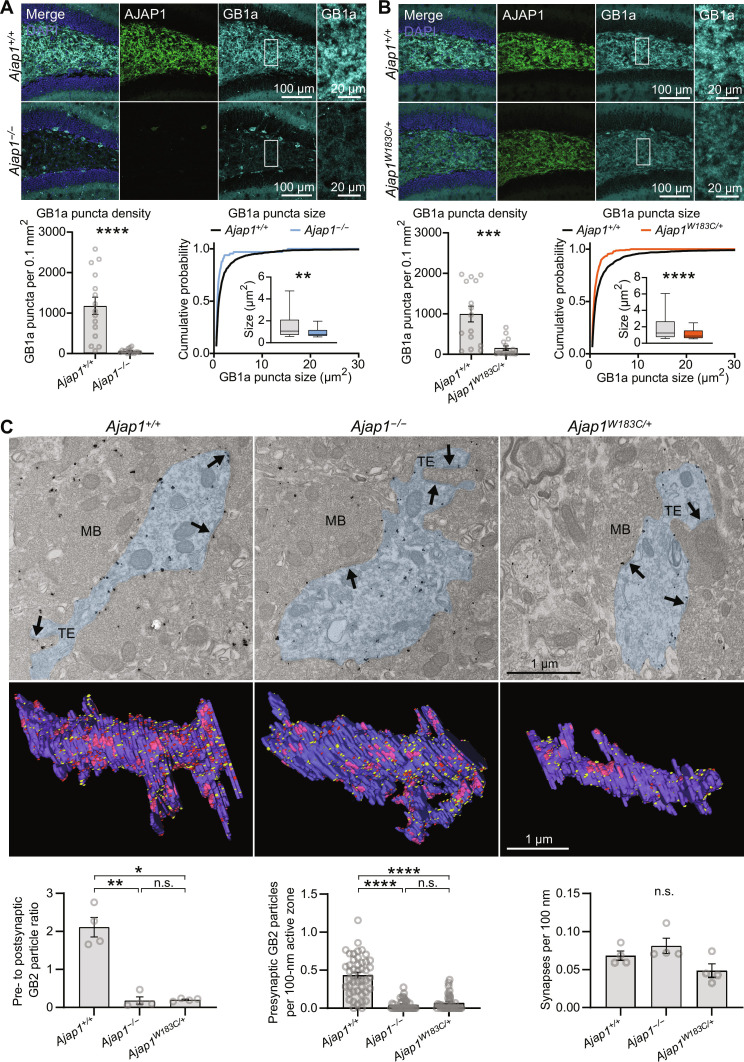
Reduction in presynaptic GBRs in *Ajap1*^−/−^ and *Ajap1*^W183C/+^ mice. (**A**) Immunolabeling in the hilus of *Ajap1^−/−^* and *Ajap1^+/+^* mice and quantification of GB1a puncta density and size in the neuropil. **P < 0.01, ****P < 0.0001, unpaired *t* test (density), Kolmogorov-Smirnov test (size), *n* = 15 sections per genotype (density), *n* = 1267 puncta (*Ajap1^+/+^*), *n* = 66 puncta (*Ajap1^−/−^*), five mice per genotype. Box plots 25th to 75th percentiles, whiskers 10th to 90th percentiles, and line indicates median. (**B**) Immunolabeling in *Ajap1*^W183C/+^ and *Ajap1^+/+^* mice and quantification of GB1 puncta density and size. ***P < 0.001, ****P < 0.0001, Mann-Whitney test (density), Kolmogorov-Smirnov test (size), *n* = 15 sections per genotype (density), *n* = 1818 puncta (*Ajap*^+/+^), *n* = 291 puncta (*Ajap1*^W183C/+^), five mice per genotype. Box plots as in (A). (**C**) Electron microscopic quantification of GBRs at putative granule cell/HMC synapses in the hilus apex using pre-embedding immunogold labeling. Top: GB2 labeling in pre- and postsynaptic elements at asymmetrical synapses (arrows). Blue area indicates HMC dendrites. MB, mossy fiber bouton; TE, thorny excrescences. Middle: Serial section reconstructions of HMC dendrites showing pre- (red) and postsynaptic (yellow) GB2 particles (active zones in pink). Bottom: *Ajap1*^−/−^ and *Ajap1*^W183C/+^ mice contain fewer immunogold particles at presynaptic elements, resulting in a significantly decreased pre- to postsynaptic particle ratio. GB2 particle density in active zones is lower in *Ajap1*^−/−^ and *Ajap1*^W183C/+^ versus *Ajap1^+/+^* mice. Synapse density (active zones per length of postsynaptic membrane) is similar among genotypes. n.s., *P* > 0.05, *P < 0.05, **P < 0.01, ****P < 0.0001, Welch’s ANOVA and Dunnett’s T3 multiple comparisons test (GB2 particle ratio); Kruskal-Wallis test and Dunn’s multiple comparisons test (GB2 particle density active zones); Kruskal-Wallis test (synapse density); *n* = 4 dendritic segments from two mice per genotype (GB2 particle ratio and synapse density), *n* = 35 to 45 active zones from two mice per genotype (GB2 particle density in active zones).

The data from the coculture assay suggest that lack of AJAP1 in neurons impairs trans-synaptic recruitment of presynaptic GB1a/2 receptors. Immunohistochemistry analysis of *Ajap1*^−/−^ mice further supports this finding. Specifically, we observed a significant reduction in the density of GB1a puncta in the hilus of the dentate gyrus (Fig. 6A). In addition, a decrease in the size of GB1a puncta suggested a decreased clustering of presynaptic GB1a protein. Similarly, there was a significant reduction in GB2 immunolabeling in the hilus of *Ajap1*^−/−^ mice, although GB2 labeling is not exclusive to presynaptic GBRs (fig. S4A). Notably, the reduction in density and size of GB1a puncta was more pronounced compared to GB2 puncta. We observed no significant changes in the number of HMCs, PSD-95 puncta, or VGluT1 puncta in the hilus of *Ajap1*^−/−^ mice, which supports that AJAP1 lacks developmental or synaptogenic properties (fig. S5, A and B). AJAP1 immunostaining is high in Purkinje cells of the cerebellum. Consequently, we observed a significant decrease in the density of GB1a puncta in the molecular layer of the cerebellum of *Ajap1*^−/−^ mice, consistent with the loss of presynaptic GBRs at synapses on Purkinje cell dendrites (fig. S6A). AJAP1 immunostaining is low in the CA1 region of the hippocampus and absent in the medial habenula (Fig. 2, A and B). *Ajap1*^−/−^ mice do not exhibit a significant reduction in the density of GB1a puncta in these areas (fig. S6, B and C). Similarly, earlier electrophysiological experiments revealed no significant decrease in presynaptic GBR-mediated inhibition at CA1 synapses of *Ajap1*^−/−^ mice (*9*). While AJAP1 may not be essential for the expression of presynaptic GBRs, its presence in the postsynapse appears to capture GBRs at the presynaptic site contingent on its postsynaptic abundance.

Heterozygous *Ajap1*^W183C/+^ mice, mimicking the monoallelic p.(W183C) patient genotype, showed a significant decrease in the density and size of GB1a and GB2 puncta in the hilus, similar to *Ajap1*^−/−^ mice (Fig. 6B and fig. S4B). Similarly, both Manders’ colocalization coefficient and Pearson’s correlation coefficients reveal a significantly reduced colocalization of AJAP1 and GB1a proteins in *Ajap1*^W183C/+^ mice compared to *Ajap1*^+/+^ control mice (fig. S4C). The immunohistochemistry data thus support that AJAP1 protein from the standard *AJAP1* allele of *Ajap1*^W183C/+^ mice only retains a fraction of GB1a protein at synapses. To directly assess presynaptic GBR levels, we conducted pre-embedding immunogold labeling experiments using anti-GB2 antibodies in the hilus of the dentate gyrus (anti-GB1a antibodies were not suitable for immunogold labeling). Synapses between granule cells and HMCs are abundant in the apex of the hilus. In WT mice, GB2 immunogold particles labeled both pre- and postsynaptic elements of asymmetrical synapses between mossy fiber boutons and putative HMC dendrites with thorny excrescences (Fig. 6C). In *Ajap1*^−/−^ and *Ajap1*^W183C/+^ mice, we found less GB2 particles in presynaptic elements but a similar density of synapses as in WT mice. The ratio of GB2 immunogold particles in pre- versus postsynaptic elements in *Ajap1*^+/+^, *Ajap1*^−/−^, and *Ajap1*^W183C/+^ mice was determined to be 2.11, 0.18, and 0.20, respectively. Accordingly, there were significantly fewer pre- than postsynaptic GB2 particles in *Ajap1*^−/−^ and *Ajap1*^W183C/+^ mice but not in *Ajap1*^+/+^ mice (fig. S7). These ultrastructural findings provide compelling evidence for a decrease in presynaptic GBRs in *Ajap1*^−/−^ and *Ajap1*^W183C/+^ mice at the synapses between granule cells and HMCs.

**Fig. 7.**
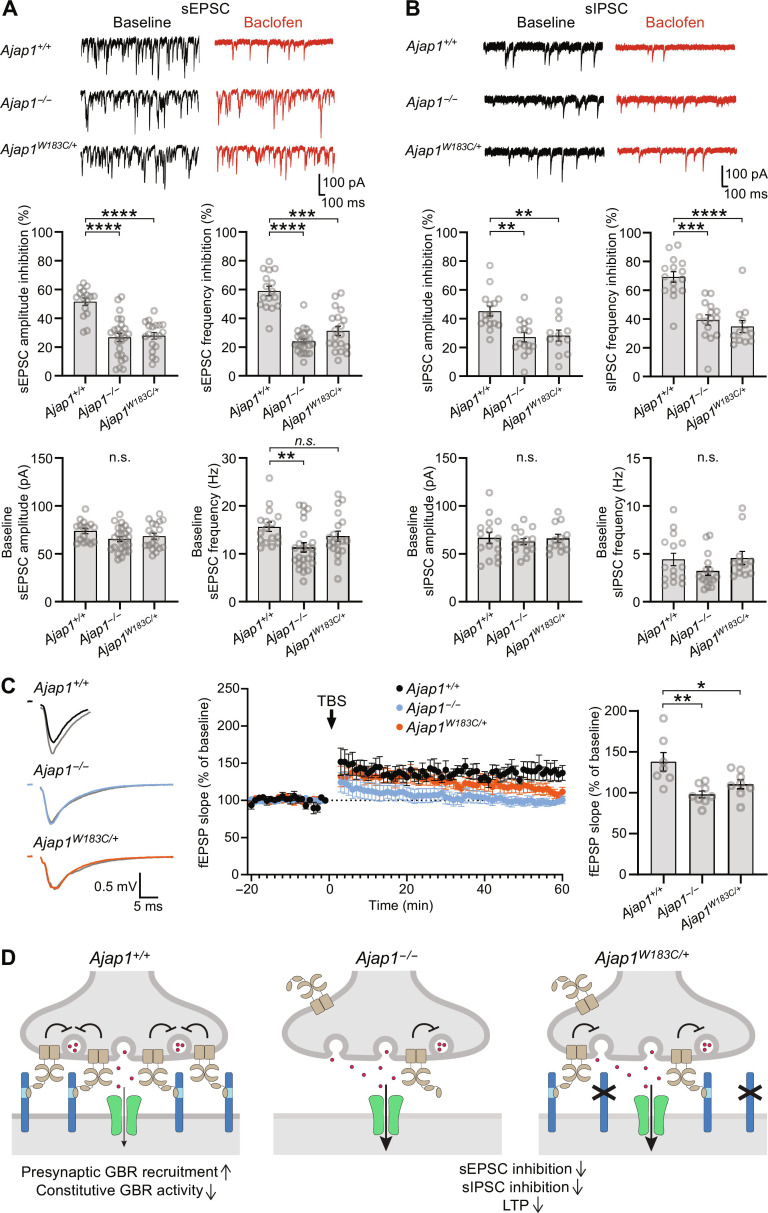
Reduced presynaptic GBR inhibition in *Ajap1^−/−^* and *Ajap1*^W183C/+^ mice. (**A**) Baclofen-induced inhibition of sEPSC amplitude and frequency in HMCs, with representative traces and bar graphs depicting quantitative analysis. n.s., *P* > 0.05, **P < 0.01, ***P < 0.001, ****P < 0.0001, one-way ANOVA, Tukey’s multiple comparisons test (amplitude); Kruskal-Wallis test, Dunn’s multiple comparisons test (frequency); *n* = 16 to 24 cells from ≥3 mice per genotype. (**B**) Baclofen-induced inhibition of sIPSC amplitude and frequency in HMCs, with representative traces and bar graphs depicting quantitative analysis. n.s., *P* > 0.05, **P < 0.01, ***P < 0.001, ****P < 0.0001, one-way ANOVA, Tukey’s multiple comparisons test (amplitude); Kruskal-Wallis test, Dunn’s multiple comparisons test (frequency); *n* = 12 to 15 cells from ≥3 mice per genotype. Combined traces from *Ajap1^+/+^* littermates of *Ajap1^−/−^* and *Ajap1*^W183C/+^ mice were used for statistical analysis of sEPSCs and sIPSCs. (**C**) Time course of fEPSP slopes (mean ± SEM) from hippocampal slices of *Ajap1^−/−^* (blue, *n* = 8 slices from six mice), *Ajap1*^W183C/+^ (orange, *n* = 8 slices from three mice), and *Ajap1*^+/+^ mice (black, *n* = 7 slices from five mice). Arrow indicates the time of theta-burst stimulation (TBS). Sample traces of fEPSPs before (black, blue, and orange) and after (gray) TBS are shown. *Ajap1*^+/+^ littermates of *Ajap1^−/−^* and *Ajap1*^W183C/+^ mice were pooled. Bar graph shows fEPSP slope 60 min after LTP induction normalized to baseline for the different genotypes. *P < 0.05, *P < 0.01, one-way ANOVA, Tukey’s multiple comparisons test. (**D**) Monoallelic AJAP1-W183C patient variant induces synaptopathy. Left: AJAP1 trans-synaptically recruits presynaptic GBRs by interacting with GB1a. NAM activity of AJAP1 limits constitutive GBR activity at high receptor density. Middle: Complete AJAP1 loss decreases presynaptic GBRs. Right: AJAP1-W183C fails to bind GB1a, reducing presynaptic GBRs. The reduction in presynaptic GBRs induced by the complete lack of AJAP1 or the AJAP1-W183C variant disinhibits neurotransmitter release and impairs synaptic plasticity.

Activation of presynaptic GBRs suppresses Ca^2+^ channel activity and inhibits neurotransmitter release (*16*). Consistent with the loss of presynaptic GBRs in *Ajap1*^−/−^ and *Ajap1*^W183C/+^ mice, we observed a significant reduction in the baclofen-mediated inhibition of spontaneous excitatory postsynaptic current (sEPSC) and inhibitory postsynaptic current (sIPSC) frequencies and amplitudes in HMCs in acute hippocampal slices (Fig. 7, A and B). Baseline sEPSC and sIPSC frequencies and amplitudes were generally normal, except for a significant reduction in baseline sEPSC frequency in *Ajap1*^−/−^ mice (Fig. 7, A and B). The paired-pulse ratio (PPR), which represents the ratio of the amplitude of the second postsynaptic response to that of the first, is inversely related to the probability of vesicular release and is commonly used to assess release probability (*34*–*36*) [but see also (*37*)]. We examined EPSCs in HMCs evoked by paired-pulse stimulation of the CA3 stratum lucidum at 10- and 100-ms interstimulus intervals (fig. S8A). Baclofen at a concentration of 100 μm equally inhibited the first EPSC amplitude in *Ajap1*^+/+^ and *Ajap1*^W183C/+^ mice (*Ajap1*^+/+^: 49.0 ± 5.2%, *Ajap1*^W183C/+^: 49.5 ± 5.8%, one-sample *t* test). We observed no significant change in the PPRs in either genotype at a 100-ms stimulation interval in the presence of baclofen (fig. S8B). However, when HMC synapses were stimulated at a 10-ms interstimulus interval, the PPR in the presence of baclofen showed a small but significant increase in *Ajap1*^+/+^ mice, while no significant change was observed in *Ajap1*^W183C/+^ mice (fig. S8B). The increased PPR observed in the presence of baclofen in *Ajap1*^+/+^ mice supports that HMC synapses in these mice exhibit a lower release probability compared to *Ajap1*^W183C/+^ mice. Loss of presynaptic GBRs in *GB1a*^−/−^ mice impaired long-term potentiation (LTP), a synaptic plasticity phenomenon thought to underlie memory formation (*17*). Consistent with this earlier finding, recordings of field excitatory postsynaptic potentials (fEPSP) in the CA1 stratum radiatum upon Schaffer collateral stimulation revealed impaired LTP in hippocampal slices from *Ajap1*^−/−^ and *Ajap1*^W183C/+^ mice (Fig. 7C).

The 4-year-old patient carrying the de novo pTrp183Cys variant experienced recurrent convulsions at a young age, with seizure-free intervals lasting several months while using antiepileptic medications (Table 1). The seizures intensity decreased with age, and the electroencephalogram (EEG) displayed no other anomalies. In *GB1a*^−/−^ mice, the lack of presynaptic GBRs leads to strong epileptiform activity (*38*). To investigate whether the partial loss of presynaptic GBRs in *Ajap1*^W183C/+^ mice affects EEG patterns or triggers seizures, we examined EEG activity in the parietal region during wakefulness, sleep, and after sleep deprivation. The results showed that EEG and electromyography (EMG) activities of *Ajap1*^W183C/+^ and *Ajap1*^+/+^ mice were similar during wakefulness, nonrapid eye movement sleep, and rapid eye movement sleep, both under baseline conditions and after sleep deprivation (fig. S9A). The EEG power spectral density profiles during different vigilance states and after sleep deprivation were similar between *Ajap1*^W183C/+^ and *Ajap1*^+/+^ mice (fig. S9B). Furthermore, the proportions of different vigilance states, transitions between states, and episode durations were comparable between *Ajap1*^W183C/+^ and *Ajap1*^+/+^ mice during baseline and after sleep deprivation (fig. S9C). These findings indicate that *Ajap1*^W183C/+^ mice with a partial deficit in presynaptic GBRs exhibited EEG patterns comparable to *Ajap1*^+/+^ mice during adulthood. However, we cannot rule out the possibility of seizures occurring at a younger age or with several days between them.

## DISCUSSION

### Trans-synaptic recruitment and allosteric modulation of presynaptic GBRs by AJAP1

Proteomic studies revealed that AJAP1 interacts with the N-terminal sushi domain of the GB1a subunit (*9*, *10*). Our data now show that AJAP1 is a postsynaptic protein that recruits presynaptic GB1a/2 receptors through a trans-synaptic interaction. Previous studies demonstrated that amyloid β precursor protein (APP) also binds to the sushi domain of GB1a/2 receptors and facilitates the trafficking of receptors in axons (*9*). We propose that AJAP1 may sequester axonal GB1a/2 receptors away from APP, given AJAP1’s affinity for the sushi domain that is more than 100-fold higher than that of APP (*9*). Trans-synaptic interactions with AJAP1 may contribute to the sushi domain–dependent stabilization of GB1a/2 receptors at synapses (*9*, *39*). We show that in cell-based in vitro assays, AJAP1 acts as a NAM and reduces constitutive GBR activity. Constitutive GBR activity has been observed when receptors are expressed at high density in transfected cells (*33*). However, it remains unclear to what extent GBRs exhibit constitutive activity in vivo, where receptor density is much lower. Nevertheless, NAM activity of AJAP1 may prevent GABA-independent suppression of neurotransmitter release at synapses with a particularly high recruitment of presynaptic GBRs. NAM activity of AJAP1 increases the effective concentration of GABA required to activate GBRs, favoring receptor activation by the high concentrations of synaptically released GABA. A comparable trans-synaptic regulation was observed with group III metabotropic glutamate receptors (mGluRs). These receptors undergo interactions with the postsynaptic adhesion molecules Extracellular Leucine Rich Repeat And Fibronectin Type III Domain Containing 1 (ELFN1) and ELFN2, which act as NAMs of mGluRs in heterologous cells (*40*–*42*). It is noteworthy that ELFN and AJAP1 exhibit no sequence homology, showing that trans-synaptic regulation of Family 3 G protein–coupled receptors evolved independently.

### p.(W183C) exhibits a loss-of-function phenotype and induces synaptic dysfunctions

Thus far, no clinical significance had been established for *AJAP1* variants. Now, we have identified several individuals with *AJAP1* variants who have epilepsy and/or neurodevelopmental phenotypes. Characterization of the p.(W183C) variant in cell-based functional assays showed that it exhibits a loss-of-function phenotype. Specifically, the p.(W183C) variant disrupts the interaction between AJAP1 and the sushi domain of GB1a, consistent with the recognition that disease-causing variants often target protein-protein interfaces (*43*).

To provide additional evidence supporting the clinical relevance of p.(W183C), we generated heterozygous *Ajap1*^W183C/+^ mice and analyzed them in conjunction with *Ajap1*^−/−^ mice. In pre-embedding immunogold labeling experiments, we observed a significant reduction in presynaptic GB1a/2 receptors at HMC synapses in both *Ajap1*^W183C/+^ and *Ajap1*^−/−^ mice. The total protein levels of GBR subunits in *Ajap1*^−/−^ mice do not show an overt decrease (*9*), suggesting that in the absence of AJAP1, presynaptic GBRs are redistributed rather than degraded. Previous studies have shown that neurexins are required for the localization and function of presynaptic GBRs (*44*). However, neurexins do not prevent the loss of presynaptic GBRs in *Ajap1*^W183C/+^ and *Ajap1^−/−^* mice, indicating that neurexins may primarily affect the global organization of the active zone rather than the specific localization of GBRs (*45*, *46*). Consistent with the loss of presynaptic GBRs observed in ultrastructural studies, synapses at HMCs of *Ajap1*^W183C/+^ and *Ajap1^−/−^* mice exhibit reduced baclofen-mediated inhibition of GABA and glutamate release and impaired LTP. Immunogold labeling and electrophysiology indicate a comparable loss of presynaptic GBRs in *Ajap1*^W183C/+^ and *Ajap1*^−/−^ mice, which might hint at a dominant-negative effect of the p.(W183C) variant. Notably, *GB1a*^−/−^ mice, in which the initiation codon for GB1a transcripts in the *Gabbr1* gene has been mutated to a stop codon, similarly exhibit a lack of presynaptic GBRs and impaired LTP (*17*). In *GB1a*^−/−^ mice, impaired LTP was attributed to the saturation of LTP caused by excess glutamate release, a mechanism that could similarly contribute to the impairment of LTP in *Ajap1*^W183C/+^ and *Ajap1*^−/−^ mice. However, lack of AJAP1 in these mice also disinhibits GABAergic terminals, potentially leading to increased postsynaptic inhibition and, consequently, the impairment of LTP (*47*). *GB1a*^−/−^ mice additionally exhibit epileptiform activity (*38*). In contrast, no EEG alterations were observed in *Ajap1*^W183C/+^ mice, which is similar to individual 1 carrying the p.(W183C) variant. While individual 1 experiences occasional seizures, the baseline EEG pattern remains normal.

Overall, our data support that AJAP1 enables the fine-tuning of presynaptic inhibition levels in a neuron-specific manner. Notably, the widespread expression of AJAP1 suggests its involvement in regulating presynaptic GBR inhibition at many synapses in the brain. On the basis of functional deficits in cellular systems and the observed synaptic dysfunctions in *Ajap1*^W183C/+^ mice, we classified p.(W183C) as pathogenic.

### Estimated pathogenic risk of additional AJAP1 variants

In cell-based assays, we observed no functional deficits for the p.(P242S) variant of individual 2, suggesting that this variant is benign. On the basis of this result, individual 2 underwent additional testing, which revealed an increased interferon signature. This is more in line with a possible Aicardi-Goutières syndrome, though no concomitant gene variants have been identified. We subsequently identified an additional individual carrying the p.(P242S) variant who presented with chronic obstructive pulmonary disease, peripheral vascular disease, and small stature. Furthermore, we also identified two unrelated individuals with a p.(P242T) variant: one afflicted by central core disease and the other a healthy parent of a child lacking this variant. The absence of typical clinical phenotypes associated with other *AJAP1* variants supports the benign nature of the p.(P242S) and p.(P242T) variants.

If the p.(I271Ffs*24) allele of individual 3 indeed results in the production of a secreted protein, as analyzed in our experiments with AJAP1-Δ273–412, we anticipate that it may induce similar synaptic dysfunctions as p.(W183C). However, p.(I271Ffs*24) could equally trigger high levels of nonsense-mediated mRNA decay and lead to haploinsufficiency. Data from the Genome Aggregation Database (gnomAD v2.1.1) suggest that *AJAP1* is likely to be haploinsufficient with 15.5 loss-of-function variants expected and none being observed, giving *AJAP1* a pLI score of 0.99 and a Loss-Of-Function Observed/Expected Upper Bound Fraction (LOEUF) score of 0.19. Similarly, *AJAP1* has a pHaplo score of 0.99, suggesting that it is likely to be haploinsufficient (*48*, *49*). We therefore classified p.(I271Ffs*24) as likely pathogenic. However, we note that individual 3 also carries a de novo *BRD4* variant that is likely responsible for the ocular phenotypes and could potentially cause or contribute to neurodevelopmental phenotypes and short stature (*50*–*52*).

Heterozygous *AJAP1* deletion in individual 4 likely leads to haploinsufficiency and is therefore likely pathogenic. Individual 4 carries a second deletion of exons 4 to 6 of *CAMTA1*, which is predicted to produce an in-frame deletion of 92 amino acids. *CAMTA1* may therefore contribute to the spectrum of neurobiological phenotypes of individual 4 (*53*, *54*)*.* It is difficult to predict whether and how the heterozygous *AJAP1* splice variant in individual 5 affects AJAP1 functions.

In conclusion, while genomic techniques have revealed numerous gene variants and deletions associated with diseases, identifying the underlying mechanisms often proves challenging. Our study contributes to the growing recognition that understanding the composition and structure of native protein complexes provides mechanistic explanations for how variants in seemingly unrelated proteins can give rise to similar clinical manifestations (*43*, *55*, *56*).

## MATERIALS AND METHODS

### Genetic analysis

Individuals 1 to 5 were evaluated at their respective clinical institutions, and their *AJAP1* variants were identified by clinically based exome sequencing or chromosomal microarray analysis. They were accrued into this study with the help of GeneMatcher (*20*). Informed consent was obtained, and individuals 1, 2, and 4 were enrolled in protocol H-42409 approved by the Institutional Review Board of Baylor College of Medicine. Anonymized information for individual 5 is being presented as allowed by protocol H-47546 approved by the Institutional Review Board of Baylor College of Medicine. Individual 3 was initially enrolled in the Disorders of Cerebral Development: A Phenotypic and Genetic Analysis Study at the Brain Development Research Program of the University of California, San Francisco (UCSF). The study protocol was approved by the Institutional Review Boards of UCSF, and the enrolled individual had informed consent provided by the parent/guardian.

### Plasmids

Mouse AJAP1 and AJAP1-SDBM (I181, W183, G184, P185, and T186 mutated to alanine) (*9*) coding sequences containing a C-terminal HA tag were inserted into the pCI vector upstream of mCherry, which generates AJAP1-mCherry fusion proteins. AJAP1-ΔCTD-mCherry was created by deleting the C-terminal 103 residues of AJAP1 (*19*). AJAP1-BLSM-mCherry (Y352, Y370, Y381, L397, and I398 mutated to alanine) was generated by introducing an 816-bp DNA fragment containing the mutations (GenScript Biotech) into pCI-AJAP1-mCherry. AJAP1 variants (W183C, P244S, and Δ273–412) were generated by site-directed mutagenesis of pCI-AJAP1-mCherry. For surface expression experiments, a FLAG epitope was inserted after K115 in the extracellular domain of AJAP1. GN-AJAP1-mCherry and GN-AJAP1-SDBM-mCherry (containing superfolder GFP1–10 (44967, Addgene) flanked by G-S linkers after AJAP1-R170) were generated using the insert as a polymerase chain reaction primer with pCI-AJAP1-mCherry or pCI-AJAP1-SDBM-mCherry as templates. GC-GB1a was generated by replacing the myc-tag of pCI-myc-GB1a (*57*) with superfolder GFP11 (no. 44968, Addgene). FLAG-GB1a (*58*), FLAG-GB2 (*9*) and TagBFP (*59*) were published earlier. For coimmunoprecipitation experiments, mCherry-tagged full-length gephyrin (68820, Addgene) was used.

### Immunoblots, immunoprecipitation, and HEK293T cell immunocytochemistry

Immunoblots, immunoprecipitation, and immunochemistry were performed as described (*9*, *18*). AJAP1 immunoprecipitation and detection were with sheep anti-AJAP1 antibody (AF7970, R&D Systems) and horseradish peroxidase (HRP)–coupled anti-sheep antibody (61–8620, Thermo Fisher Scientific). Gephyrin was detected with mouse anti-gephyrin antibody (147 111, Synaptic Systems) and HRP-coupled anti-mouse antibody (NA931V, GE Healthcare). SuperSignal Pico (34580, Thermo Fisher Scientific) and Femto chemiluminescence detection kits (34094, Thermo Fisher Scientific) were used for visualization on immunoblots. Band intensities were quantified by ImageJ software, as described (*9*).

### Isothermal titration calorimetry

Experiments were carried out using a microcalorimeter (Microcal ITC200, GE Healthcare, Chicago, USA) at 25°C with a stirring speed of 600 rpm in a buffer containing 20 mM NaP_i_ (pH 7.4). For titration, 100 μM hAJAP17 or 300 μM hAPP17-W183C were injected (first injection 0.5 μl, followed by 25 injections of 1.5 μl) into the sample cell containing 10 or 30 μM purified recombinant sushi domain protein (*10*), respectively. Peptide versus buffer control measurements were subtracted from peptide versus sushi domain measurements. Analysis was performed with Microcal ITC200 Origin software using a one-site binding model.

### Fluorescence polarization

Binding affinities were determined in a flat bottom black 384-well plate (Corning Life Science) using a Safire2 plate reader (Tecan). Experiments were carried out in buffer containing 20 mM NaP_i_ (pH 6.8), 50 mM NaCl, 0.5 mM EDTA, and 1% bovine serum albumin at 25°C. Fluorescence measurements were at the excitation/emission wavelengths of 530/580 nm. The Z-factor was adjusted to maximum fluorescence and the G-factor was calibrated to give an initial milli-polarization of 20. Saturation binding or competition experiments were performed using TMR-ETEFIAWGPTGDEEALE-NH_2_. For saturation binding, sushi domain protein was measured at 12 different concentrations (0.73 to 1500 nM) by making 1:1 dilutions. Experiments were in triplicate and the data fitted to a one-site binding model. For competition experiments, a preformed complex of sushi domain/TMR-ETEFIAWGPTGDEEALE-NH_2_ was outcompeted with unlabeled peptides at 12 different concentrations ranging from 0.03 to 62.5 μM (hAJAP17-W183C, mAJAP17-W183C, and mAJAP17-scrambled) or 0.006 to 6.25 μM (hAJAP17, mAJAP17). Experiments were performed at least in triplicate, and the data fitted to a sigmoidal dose-response curve using GraphPad Prism 7. *K*_i_ values were calculated as described (*60*).

### Trans-cellular fluorescence complementation

Two populations of HEK293T cells were separately transfected with the following constructs: (i) AJAP1-GFP1–10, AJAP1-SDBM-GFP1–10, or WT AJAP1 as a control; (ii) GFP11-GB1a, GB2, and TagBFP. After 12 hours, cells were washed with Opti-MEM and detached with 0.25% trypsin containing EDTA (25200056, Gibco). Trypsin was removed by centrifugation at 230*g*, and cells resuspended in Dulbecco’s modified Eagle’s medium (DMEM) containing 10% fetal calf serum (FCS). Equal numbers of cells from each population were combined, mixed, and plated at 85,000 cells/cm^2^ on poly-l-lysine–coated (0.02 mg/ml; P9155, Sigma-Aldrich) glass coverslips in DMEM containing 10% FCS at 37°C/5% CO_2_. Mixed cell cultures were maintained for 24 hours until processing for immunofluorescence analysis.

### Mice

*Ajap1*^−/−^ (*9*), *Ajap1*^HA/HA^, and *Ajap1*^W183C/+^ mice were kept in the C57BL/6 J background. *Ajap1*^W183C/+^ mice were generated by CRISPR-Cas9–mediated disruption of exon 2 and homology-directed repair. Oocytes were injected with single-stranded oligodeoxynucleotide (ssODN) and a spCas9/gRNA ribonucleoprotein complex targeting the 5′-CTCATCCCCCGTAGGCCCCCAGG-3′ sequence on the antisense strand. Homology-directed repair using the ssODN template introduced a C-to-A mutation at position 517 (silent mutation to disrupt the protospacer adjacent motif) and a G-to-C mutation at position 520 of exon 2. *Ajap1^HA/HA^* mice were generated by CRISPR-Cas9–mediated disruption of exon 5 and homology-directed repair inserting a HA tag at the C terminus of AJAP1. Oocytes were injected with an ssODN including the 5′-TACCCTTACGACGTTCCAGACTACGCT-3′ HA sequence and a spCas9/gRNA ribonucleoprotein complex targeting the 5′-AAAGCCTTCGGCCAGTCAGCAGG-3′ sequence on the antisense strand. HA insertion disrupted the protospacer sequence. Mice were kept in a 12-hour light/dark cycle with water and food ad libitum. All mouse experiments were performed in accordance with the guidelines of the Veterinary Offices of Basel-Stadt (license no. 1897_31476) and Lausanne (license no. 3524).

### Proteomic analysis

Membrane-enriched protein fractions were prepared from frozen brains of adult *Ajap1*^HA/HA^ and WT mice (three mice each) as described (*61*). For each affinity purification, 1 mg of membrane proteins was solubilized in 1 ml of CL-91 (Logopharm GmbH, Germany) for 30 min on ice. After clearing by ultracentrifugation (135,000*g*, 10 min) solubilisates were incubated with 10 μg of immobilized (Protein A Dynabeads, Thermo Fisher Scientific) anti-HA antibodies (Ab1: rat polyclonal anti-HA, 47877600, Roche; Ab2: mouse monoclonal anti-HA, 26183, Thermo Fisher Scientific) for 2 hours on ice. After washing with 0.5 ml of CL-91, proteins were eluted with 10 μl of Laemmli buffer (DTT added after elution). Proteins were run into SDS–polyacrylamide gel electrophoresis, silver stained lanes cut into two pieces; and in-gel digested with sequencing grade trypsin (Promega GmbH, Walldorf, Germany). Vacuum-dried peptides were dissolved in 0.5% (v/v) trifluoroacetic acid. Appropriate amounts were loaded onto trap columns (C18 PepMap100, 5-μm particles, Thermo Fisher Scientific) with 0.05% trifluoroacetic acid and separated on C18 reversed-phase columns (SilicaTip emitters, 75-μm inside diameter, 8-μm tip, New Objective Inc., Littleton, USA, manually packed 11 to 12 cm (Orbitrap Elite mass spectrometer) with ReproSil-Pur ODS-3, 3-μm particles, Dr. A. Maisch HPLC GmbH, Ammerbuch-Entringen, Germany; flow rate: 300 nl/min) using an UltiMate 3000 RSLCnano HPLC systems (Thermo Fisher Scientific GmbH, Dreieich, Germany). Gradients were built with eluent A (0.5% (v/v) acetic acid in water) and eluent B (0.5% (v/v) acetic acid in 80% (v/v) acetonitrile/20% (v/v) water): 5 min 3% B, 60 min from 3% B to 30% B, 15 min from 30% B to 99% B, 5 min 99% B, 5 min from 99% B to 3% B, 15 min 3% B (Orbitrap Elite mass spectrometer). Eluting peptides were electrosprayed at 2.3 kV (positive polarity) via Nanospray Flex ion sources into an Orbitrap Elite mass spectrometer [collision-induced dissociation fragmentation of the 10 most abundant at least doubly charged new precursors per scan cycle) and analyzed with the following settings: scan range 370 to 1700 mass/charge ratio, full MS resolution 240,000, dd-MS2 resolution “normal” (ion trap). Liquid chromatography–tandem mass spectrometry RAW files were converted into peak lists (Mascot generic format, mgf) with ProteoWizard msConvert (https://proteowizard.sourceforge.io/). All peak lists were searched with Mascot Server (Matrix Science Ltd., London, UK) against a database containing all mouse, rat, and human entries of the UniProtKB/Swiss-Prot database (peptide mass tolerance ±5 ppm; fragment mass tolerance 0.8 Da). One missed trypsin cleavage and common variable modifications were accepted. A label-free quantification procedure (*62*) was used to determine specific copurification of proteins in anti-HA affinity purifications. Abundances of all proteins detected in eluates obtained from *Ajap1*^HA/HA^ and WT control samples were used to build abundance ratios (target normalized ratio).

### Immunohistochemistry

Two- to 4-month-old mice were anaesthetized with ketamine (200 mg/kg i.p.; Ketalar, Pfizer) and xylazine (16 mg/kg i.p.; Rompun, Bayer), transcardially perfused with ice-cold oxygenated artificial cerebrospinal fluid (ACSF, pH 7.4), brains dissected on ice and fixed with 4% paraformaldehyde in phosphate-buffered saline (PBS) for 2 hours at 4°C. Brains were dehydrated with 30% sucrose in PBS for 24 hours at 4°C, 50-μm-thick coronal or sagittal sections cut with a cryostat and sections stored in 37.5% ethylene glycol and 1 M glucose in 50 mM phosphate buffer at −20°C. Free-floating sections were washed with PBS and incubated in blocking solution [2% normal donkey serum (NDS) and 0.2% Triton X-100 in PBS] for 1 hour at room temperature (RT). Sections were incubated overnight at 4°C in blocking solution containing primary antibodies, washed in PBS, and incubated in 2% NDS in PBS containing secondary antibodies for 1 hour at RT. DAPI (4′,6-diamidino-2-phenylindole) was applied during secondary antibody incubation at a final concentration of 2 μg/ml. Sections were washed in PBS and mounted on gelatine-coated slides with Fluorescence Mounting Medium (S3023, Agilent). The following primary antibodies were used: Sheep anti-AJAP1 (AF7970, R&D Systems), rabbit anti-GluA2 (AB1768-I, Merck), mouse anti-Bassoon (VAPS003, Stressgen), rabbit anti-Bassoon (141 003, Synaptic Systems), chicken anti-MAP2 (ab5392, Abcam), mouse anti–Ankyrin-G (75–147, Neuromab), mouse anti–PSD-95 (124 011, Synaptic Systems), rabbit anti-VGluT1 (135 303, Synaptic Systems), mouse anti-gephyrin (147 021, Synaptic Systems), rabbit anti-VGAT (131 003, Synaptic Systems), guinea pig anti-GB2 (322 205, Synaptic Systems), mouse anti-GB1a (*63*), rabbit anti-Synapsin1/2 (106 002, Synaptic Systems), mouse anti-β-TubulinIII (T8660, Sigma-Aldrich), mouse anti-FLAG (F1804, Sigma-Aldrich), mouse anti-Calbindin1 (300, Swant). The following secondary antibodies were used: Donkey anti-mouse IgG (H+L) DyLight 405 (715–475-150, Jackson ImmunoResearch), donkey anti-mouse IgG (H+L) Alexa Fluor 488 (A-21202, Thermo Fisher Scientific), donkey anti-mouse IgG (H+L) Alexa Fluor 555 (A-31570, Thermo Fisher Scientific), donkey anti-mouse IgG (H+L) Alexa Fluor 647 (A-31571, Thermo Fisher Scientific), donkey anti-rabbit IgG (H+L) DyLight 405 (711–475-152, Jackson ImmunoResearch), donkey anti-rabbit IgG (H+L) Alexa Fluor 647 (A-31573, Thermo Fisher Scientific), donkey anti-guinea pig IgG (H+L) Alexa Fluor 488 (706–005-148, Jackson ImmunoResearch), donkey anti-guinea pig IgG (H+L) Alexa Fluor 647 (706–605-148, Jackson ImmunoResearch), donkey anti-sheep IgG (H+L) Alexa Fluor 488 (A-11015, Thermo Fisher Scientific), donkey anti-sheep IgG (H+L) Alexa Fluor 555 (A-21436, Thermo Fisher Scientific), and donkey anti-chicken IgY (IgG) (H+L) DyLight 405 (703–475-155, Jackson ImmunoResearch).

### RNA–fluorescence in situ hybridization

Mice were anaesthetized with isoflurane (Attane, Piramal Healthcare Limited) and decapitated. Brains were dissected on ice, frozen on dry ice, stored at −80°C, and then mounted with Tissue-Tek O.C.T. Compound (4583, Sakura Finetek) in the cryostat (CM3050 S, Leica) at −20°C. Coronal or sagittal sections (20 μm thick) were cut and mounted on SuperFrost Plus slides (J1800AMNZ, Thermo Fisher Scientific). Sections were dried for 1 hour and fixed with 4% paraformaldehyde in PBS for 15 min at 4°C. Slides were dehydrated in 50%, 70%, and twice in 100% ethanol for 5 min each at RT. Protein digestion and hybridization of probes was performed with RNAscope Fluorescent Multiplex Reagent Kit (320850, ACD Bio) according to manufacturer’s instructions. Sections were mounted with ProLong Gold Antifade Mountant (P36930, Thermo Fisher Scientific). The following RNAscope probes were used: Mm-Ajap1-C3, Mm-Gria2-C2, and Mm-Calb1-C2. Final amplification steps were with Amp 4-FL C, enabling detection of AJAP1 by Alexa488 and Gria2 and Calb1 by Atto647.

### Neuronal cultures

Hippocampi were dissected from E15.5 embryos in ice-cold Hanks’ balanced salt solution (HBSS) (14170088, Gibco), digested with 0.25% trypsin (15090046, Gibco) in HBSS for 15 min at 37°C and washed with HC-MEM [10% horse serum and 0.6% glucose in minimum essential medium (MEM) containing GlutaMAX (41090028, Gibco)]. Cells were dissociated with a pipette and plated at 50′000 cells/cm^2^ on poly-l-lysine–coated (0.02 mg/ml; P9155, Sigma-Aldrich) glass coverslips in HC-MEM and incubated at 37°C/5% CO_2_. After 3 hours, the medium was replaced with Neurobasal medium (21103049, Gibco) containing 1× B27 supplement (17504044, Gibco) and 1 mM GlutaMAX (35050061, Gibco).

### HEK293T cell/neuronal coculture

HEK293T cells were transiently transfected with AJAP1 constructs. Six hours after transfection, cells were briefly washed with Opti-MEM, detached with 0.25% trypsin containing EDTA (25200056, Gibco) and centrifuged at 230*g* before they were resuspended in fresh Neurobasal medium (21103049, Gibco) and plated at a density of 13,000 cells/cm^2^ onto day in vitro 12 (DIV 12) primary hippocampal mouse cultures. HEK293T and primary hippocampal cells were cocultured for 36 hours at 37°C/5% CO_2_ until they were processed for immunocytochemistry.

### Transfection of cultured neurons and immunocytochemistry

Half of conditioned medium of primary hippocampal neurons at DIV 7 was set aside and replaced with fresh Neurobasal medium containing B27 supplement and GlutaMAX. Lipofectamine 3000 reagent (L3000001, Invitrogen) was incubated with plasmid DNA in Opti-MEM medium (31985047, Gibco) at a ratio of 1 μl per microgram of DNA for 20 min. Neurons were incubated with Lipofectamine 3000/DNA mixture for 45 min, washed with Neurobasal medium, and maintained in a 1:1 mixture of Neurobasal and conditioned medium at 37°C/5% CO_2_. For immunocytochemistry of cultured neurons, cells at DIV 21 were briefly washed with PBS, fixed with 4% paraformaldehyde in PBS for 10 min at RT, washed again with PBS and permeabilized with 0.2% Triton X-100 in PBS containing 10% NDS for 5 min. Cultures were incubated with primary antibodies diluted in PBS containing 10% NDS for 2 hours. After washing with PBS, cultures were incubated with secondary antibodies diluted in PBS for 1 hour, washed three times with PBS, and air-dried. Coverslips were mounted with Fluorescence Mounting Medium (S3023, Agilent). For subcellular trafficking experiments, transfected neurons were fixed on DIV 11 with 4% paraformaldehyde in PBS for 10 min, washed three times with PBS, air-dried, and mounted with Fluorescence Mounting Medium.

### Confocal light microscopy and image analysis

Immunocyto- and histochemical stainings were imaged with an LSM700 confocal microscope (Zeiss), using 10×, 20×, 40×, and 63× Plan-Apochromat objectives with numerical apertures of 0.45, 0.80, 1.30 and 1.40, respectively. Images were processed and quantified with ImageJ. Quantification of punctate immunolabeling was performed using a custom ImageJ macro, which included the application of a Gaussian blur with radius of 1 pixel, setting a threshold based on fluorescence intensity of dentate gyrus molecular layer to generate a binary image, and quantification of puncta density and size. Puncta smaller than 0.5 μm^2^ and larger than 100 μm^2^ were not considered. Pearson’s correlation coefficients and Manders’ colocalization coefficients were calculated on single optical sections using the JACoP plugin in ImageJ. Image analysis parameters were held constant between conditions.

### Structured illumination microscopy

Three-dimensional structured illumination microscopy (3D-SIM) was performed on a DeltaVision OMX-Blaze V4 system (Leica) equipped with solid-state lasers. Images were acquired using a Plan Apo N 60×, 1.42–numerical aperture oil immersion objective lens (Olympus) and 4 liquid-cooled scientific complementary metal-oxide semiconductor cameras (pco.edge 5.5, full frame 2560 × 2160; PCO). Exciting light was directed through a movable optical grating to generate a fine-striped interference pattern on the sample plane. The pattern was shifted laterally through five phases and three angular rotations of 60° for each z-section. The 488-, 568-, and 642-nm laser lines were used during acquisition and the optical z-sections were separated by 0.125 μm. Laser power was attenuated to 10% and exposure times were between 20 and 100 ms, and the power of each laser was adjusted to achieve optimal intensities of between 5000 and 8000 counts in a raw image of 15-bit dynamic range at the lowest laser power possible to minimize photobleaching. Multichannel imaging was achieved through sequential acquisition of wavelengths by separate cameras. Raw 3D-SIM images were processed and reconstructed using the DeltaVision OMX SoftWoRx software package (v. 7.0.0 release RC 6, GE Healthcare). The resulting size of the reconstructed images was of 1024 × 1024 pixels from an initial set of 512 × 512 raw images. The channels were aligned in the image plane and around the optical axis using predetermined shifts as measured using a target lens and the SoftWoRx alignment tool. The channels were then carefully aligned using alignment parameters from control measurements with 0.5-μm-diameter multispectral fluorescent beads (Invitrogen, Thermo Fisher Scientific).

### Transcellular SRE-luciferase assay

A pool of HEK293T-Gα_qi_ cells stably expressing Gα_qi_ was transfected with Flag-GB1a, Flag-GB2, and SRE-FLuc (*33*). A separate pool of HEK293T cells was transfected with AJAP1 constructs. Six hours after transfection, cells were detached with 0.25% trypsin containing EDTA (25200056, Gibco), collected in DMEM-GlutaMAX supplemented with 10% FBS and 2% penicillin/streptomycin, centrifuged at 230*g*, and resuspended in 1 ml of DMEM-GlutaMAX (10566016, Gibco) supplemented with FBS and penicillin/streptomycin. Equal numbers of cells from both pools were combined, mixed, and distributed into 96-well microplates (7.655 083, Greiner Bio-One) at a density of 100,000 cells per well. FLuc activity in response to GABA was determined as described (*18*). Luminescence signals were adjusted by subtracting the luminescence obtained in the absence of GBRs.

### Pre-embedding immunolabeling and electron microscopy

Pre-embedding immunolabeling was performed as previously described (*64*). Briefly, mice were perfused with 0.1 M phosphate buffer (PB) containing 4% paraformaldehyde and 0.05% glutaraldehyde. Brains were excised, and 50-μm-thick hippocampal slices were prepared and then cryoprotected via incubation steps in PB containing increasing amounts of sucrose (from 5 to 15 to 20% sucrose). Sections were then rapidly frozen in liquid nitrogen and immediately thawed three times. Freeze-thawed sections were incubated in 50 mM glycine (Sigma-Aldrich) in 50 mM tris-buffered saline (TBS) and washed in TBS. Sections were blocked in 2% BSA with 10% NGS in TBS followed by incubation in primary antibodies guinea pig anti-GB2 (1 μg/ml) in 2% BSA in TBS at 4°C for 2 days and 1.4-nm gold-conjugated secondary anti-guinea pig antibody (Nanoprobes Inc., USA) for silver intensification for 1 day at 4°C. After washing in TBS and PBS, sections were postfixed in 1% glutaraldehyde in PBS, washed with 50 mM glycine in PBS, followed by another wash in PBS. Silver intensification was performed using HQ silver enhancement kit (Nanoprobes Inc., USA). Equal drops of component A (initiator) and component B (moderator), followed by the addition of component C (activator). Sections were incubated in this solution in the dark and silver intensification reaction was stopped by adding MQ water, followed by washes in 0.1 M PB. Sections were then fixed with 1% osmium tetroxide in 0.1 M PB, counterstained in 1% uranyl acetate, and dehydrated with ethanol and propylene oxide (Sigma-Aldrich). Then, sections were flat-embedded onto silicon coated glass slides and covered with an ACLAR fluoropolymer film. The most medial regions of the hilus that do not contain any CA3 pyramidal cells were trimmed and re-embedded in resin followed by cutting of 70-nm-thick serial sections with a Leica EM UC7 ultramicrotome (Leica, Germany) and observation in a Tecnai 12 transmission electron microscope.

### EM reconstruction and quantitative analysis

Serial images were taken from the trimmed hilar regions within a few microns of the surface of ultrathin sections at X15,000 using a charge-coupled device camera (VELETA, Olympus). For the reconstruction of tentative mossy cell dendrites, 67 to 108 serial ultrathin sections containing dendrites with the thorny excrescence were used. Sequential images were aligned and stacked using the TrakEM2 program (*65*). For the quantification of immunogold particles for GB2 on these reconstructed profiles, particles along the plasma membrane of the putative mossy cell dendrites and presynaptic profiles of granule cell mossy fiber boutons were counted. Immunogold particles within 20 nm of the membranes were included in the analysis on the basis of the possible distance of the immunogold particle from the epitope (*66*). For measuring the pre- to postsynaptic particle ratio, the total number of gold particles on presynaptic membranes facing the postsynaptic target was divided by the number of gold particles on the postsynaptic membrane in segments of 7–10 serial images. For the GB2 particle density in the active zone (AZ), the number of particles in all AZs of each image was divided by the sum of the lengths (nm) of all AZs in the corresponding image and multiplied by 100. AZs were identified via the presence of a postsynaptic density, rigid alignment of pre- and postsynaptic membranes and electron density in the synaptic cleft. The density of synapses was measured by counting the number of AZs per 100 nm of postsynaptic membrane in the same segments.

### Electrophysiology

Transverse 300-μm-thick hippocampal slices of 1- to 2-month-old mice were incubated at 35°C, transferred into a recording chamber, and superfused with physiological saline: 125 mM NaCl, 25 mM NaHCO_3_, 2.5 mM KCl, 1.25 mM NaH_2_PO_4_, 1 mM MgCl_2_, 2 mM CaCl_2_, and 25 mM glucose, equilibrated with a 95% O_2_/5% CO_2_ at RT (*67*). Patch pipettes were pulled from borosilicate glass (Hilgenberg; outer diameter, 2 mm; wall thickness, 0.5 mm for somatic recordings) had resistances of ∼2 to 4 megohms when filled with internal solution. Recordings were done with a Multiclamp 700B amplifier (Molecular Devices); pipette capacitance of both electrodes was compensated to 70–90%. Voltage and current signals were filtered at 10 kHz with the built-in low-pass Bessel filter and digitized at 20 kHz using Digidata 1440A (Molecular Devices). Stimulation and data acquisition were done with pClamp10 software (Molecular Devices). Holding potential of HMCs was −70 mV. For recording sEPSCs, pipettes were filled with 120 mM KMeHSO_3_, 20 mM KCl, 2 mM MgCl_2_, 2 mM Na_2_ATP, 10 mM Hepes, and 0.1 mM EGTA). For recording sIPSCs, pipettes were filled with 110 mM KCl, 35 mM K gluconate, 10 mM EGTA, 2 mM MgCl_2_, 2 mM Na_2_ATP, 10 mM Hepes, and 1 mM QX-314. sIPSCs were recorded in presence of 50 μM CNQX and 50 μM D-APV. Baclofen (100 μM) was only applied once per hippocampal slice. Paired-pulse stimulation at 10 or 100 Hz in the stratum lucidum of CA3 was with a monopolar glass electrode (digitimer DS3) filled with ACSF. For recording evoked EPSCs, the recording pipette included 1 mM QX-314. For EPSC amplitude measurements, the baseline was defined by 2-ms duration preceding the stimulation artifacts to the peak for both EPSC_1_ and EPSC_2_. To ensure stable recordings, membrane holding currents, input resistance, and pipette series resistance were monitored throughout the recordings. fEPSP were recorded in the CA1 stratum radiatum upon stimulation of the Schaffer collaterals with a bipolar concentric platinum electrode (FCH Inc., USA) placed at the CA2/CA1 border. Glass pipettes had a 3- to 4-megohm resistance and were filled with ACSF (124 mM NaCl, 3.5 mM KCl, 1.25 mM KH_2_PO_4_, 26 mM NaHCO_3_, 10 mM d-glucose, 4 mM MgCl_2_, and 0.5 mM CaCl_2_, saturated with 95% O_2_ and 5% CO_2_). For LTP studies, baseline fEPSPs were recorded every 15 s for 20 min and presented as the average of four consecutive responses. LTP was induced by four theta-burst stimulation (TBS) trains consisting of 10 bursts (five pulses at 100 Hz) delivered with an interburst interval of 150 ms, and repeated four times every 15 s. After TBS, stimulation was paused for 3 min to omit the recording of post-tetanic potentiation. Then, basal stimulation is resumed and recorded for 60 min. LTP levels were calculated by averaging the relative responses 1 hour after LTP induction. Signals were filtered at 5 kHz (Multiclamp 700B, Molecular Devices, USA) and acquired using the software WinLTP 3.0 (Anderson and Collingridge, 2007). The slopes of the fEPSPs were obtained by linear regression over the maximum initial slope points (0.5 ms), following the fiber volley.

### Polysomnography

EEG/EMG headmounts (Pinnacle Technology Inc.) were implanted on 4- to 6-month-old male mice under isoflurane-induced anesthesia, with EMG electrodes in the neck muscle and EEG electrodes (anteroposterior, mediolateral positions given relative to bregma): parietal (−3.0 mm, +1.7 mm), common reference (−4.0 mm, −2.0 mm), and ground (+0.0 mm, −2.0 mm). EEG and EMG signals were sampled at 2000 Hz (Sirenia Acquisition, Pinnacle Technology Inc.) and analyzed as described (*38*, *68*). Power spectral density was calculated using down-sampled EEG signals (400 Hz) and normalized according to established procedures (*68*).

### Statistical analysis

Statistical analysis was conducted with GraphPad Prism 9 software. Data were tested for normality using D’Agostino-Pearson test and inferential statistics were performed as described in figure legends. *P* values <0.05 were considered statistically significant. In bar graphs, data are presented as mean ± SEM. Samples were randomly assigned to experimental conditions. Data from cell culture experiments are from at least three independent experiments (cell culture preparations).

## References

[1] S. Bharti, H. Handrow-Metzmacher, S. Zickenheiner, A. Zeitvogel, R. Baumann, A. Starzinski-Powitz, Novel membrane protein shrew-1 targets to cadherin-mediated junctions in polarized epithelial cells. Mol. Biol. Cell 15, 397–406 (2004). 14595118 10.1091/mbc.E03-05-0281 PMC307556

[2] N. Lin, C. Di, K. Bortoff, J. Fu, P. Truszkowski, P. Killela, C. Duncan, R. McLendon, D. Bigner, S. Gregory, D. C. Adamson, Deletion or epigenetic silencing of AJAP1 on 1p36 in glioblastoma. Mol. Cancer Res. 10, 208–217 (2012). 22241217 10.1158/1541-7786.MCR-10-0109 PMC3288240

[3] V. Anttila, B. S. Winsvold, P. Gormley, T. Kurth, F. Bettella, G. McMahon, M. Kallela, R. Malik, B. de Vries, G. Terwindt, S. E. Medland, U. Todt, W. L. McArdle, L. Quaye, M. Koiranen, M. A. Ikram, T. Lehtimaki, A. H. Stam, L. Ligthart, J. Wedenoja, I. Dunham, B. M. Neale, P. Palta, E. Hamalainen, M. Schurks, L. M. Rose, J. E. Buring, P. M. Ridker, S. Steinberg, H. Stefansson, F. Jakobsson, D. A. Lawlor, D. M. Evans, S. M. Ring, M. Farkkila, V. Artto, M. A. Kaunisto, T. Freilinger, J. Schoenen, R. R. Frants, N. Pelzer, C. M. Weller, R. Zielman, A. C. Heath, P. A. F. Madden, G. W. Montgomery, N. G. Martin, G. Borck, H. Gobel, A. Heinze, K. Heinze-Kuhn, F. M. K. Williams, A. L. Hartikainen, A. Pouta, J. van den Ende, A. G. Uitterlinden, A. Hofman, N. Amin, J. J. Hottenga, J. M. Vink, K. Heikkila, M. Alexander, B. Muller-Myhsok, S. Schreiber, T. Meitinger, H. E. Wichmann, A. Aromaa, J. G. Eriksson, B. Traynor, D. Trabzuni, North American Brain Expression Consortium, UK Brain Expression Consortium, E. Rossin, K. Lage, S. B. R. Jacobs, J. R. Gibbs, E. Birney, J. Kaprio, B. W. Penninx, D. I. Boomsma, C. van Duijn, O. Raitakari, M. R. Jarvelin, J. A. Zwart, L. Cherkas, D. P. Strachan, C. Kubisch, M. D. Ferrari, A. van den Maagdenberg, M. Dichgans, M. Wessman, G. D. Smith, K. Stefansson, M. J. Daly, D. R. Nyholt, D. Chasman, A. Palotie, Genome-wide meta-analysis identifies new susceptibility loci for migraine. Nat. Genet. 45, 912–917 (2013). 23793025 10.1038/ng.2676 PMC4041123

[4] M. Zhang, X. Zhou, W. Jiang, M. Li, R. Zhou, S. Zhou, AJAP1 affects behavioral changes and GABA_BR1_ level in epileptic mice. Biochem. Biophys. Res. Commun. 524, 1057–1063 (2020). 32067740 10.1016/j.bbrc.2020.02.036

[5] M. Ikeda, Y. Tomita, A. Mouri, M. Koga, T. Okochi, R. Yoshimura, Y. Yamanouchi, Y. Kinoshita, R. Hashimoto, H. J. Williams, M. Takeda, J. Nakamura, T. Nabeshima, M. J. Owen, M. C. O'Donovan, H. Honda, T. Arinami, N. Ozaki, N. Iwata, Identification of novel candidate genes for treatment response to risperidone and susceptibility for schizophrenia: Integrated analysis among pharmacogenomics, mouse expression, and genetic case-control association approaches. Biol. Psychiatry 67, 263–269 (2010). 19850283 10.1016/j.biopsych.2009.08.030

[6] B. Isidor, M. Le Cunff, M. Boceno, P. Boisseau, C. Thomas, J. M. Rival, A. David, C., Complex constitutional subtelomeric 1p36.3 deletion/duplication in a mentally retarded child with neonatal neuroblastoma. Eur. J. Med. Genet. 51, 679–684 (2008). 18672103 10.1016/j.ejmg.2008.06.004

[7] W. Jang, Y. Kim, E. Han, J. Park, H. Chae, A. Kwon, H. Choi, J. Kim, J. O. Son, S. J. Lee, B. Y. Hong, D. H. Jang, J. Y. Han, J. H. Lee, S. Y. Kim, I. G. Lee, I. K. Sung, Y. Moon, M. Kim, J. H. Park, Chromosomal microarray analysis as a first-tier clinical diagnostic test in patients with developmental delay/intellectual disability, autism spectrum disorders, and multiple congenital anomalies: A prospective multicenter study in Korea. Ann. Lab. Med. 39, 299, 310 (2019). 30623622 10.3343/alm.2019.39.3.299 PMC6340852

[8] E. M. Reinthaler, D. Lal, S. Lebon, M. S. Hildebrand, H. H. M. Dahl, B. M. Regan, M. Feucht, H. Steinbock, B. Neophytou, G. M. Ronen, L. Roche, U. Gruber-Sedlmayr, J. Geldner, E. Haberlandt, P. Hoffmann, S. Herms, C. Gieger, M. Waldenberger, A. Franke, M. Wittig, S. Schoch, A. J. Becker, A. Hahn, K. Mannik, M. R. Toliat, G. Winterer, 16p11.2 European Consortium, H. Lerche, P. Nurnberg, H. Mefford, I. E. Scheffer, S. F. Berkovic, J. S. Beckmann, EPICURE Consortium, EuroEPINOMICS Consortium, T. Sander, S. Jacquemont, A. Reymond, F. Zimprich, B. A. Neubauer, 16p11.2 600 kb Duplications confer risk for typical and atypical Rolandic epilepsy. Hum. Mol. Genet. 23, 6069–6080 (2014). 24939913 10.1093/hmg/ddu306

[9] M. C. Dinamarca, A. Raveh, A. Schneider, T. Fritzius, S. Fruh, P. D. Rem, M. Stawarski, T. Lalanne, R. Turecek, M. Choo, V. Besseyrias, W. Bildl, D. Bentrop, M. Staufenbiel, M. Gassmann, B. Fakler, J. Schwenk, B. Bettler, Complex formation of APP with GABA_B_ receptors links axonal trafficking to amyloidogenic processing. Nat. Commun. 10, 1331 (2019). 30902970 10.1038/s41467-019-09164-3 PMC6430795

[10] J. Schwenk, E. Perez-Garci, A. Schneider, A. Kollewe, A. Gauthier-Kemper, T. Fritzius, A. Raveh, M. C. Dinamarca, A. Hanuschkin, W. Bildl, J. Klingauf, M. Gassmann, U. Schulte, B. Bettler, B. Fakler, Modular composition and dynamics of native GABA_B_ receptors identified by high-resolution proteomics. Nat. Neurosci. 19, 233–242 (2016). 26691831 10.1038/nn.4198

[11] J. P. Pin, B. Bettler, Organization and functions of mGlu and GABA_B_ receptor complexes. Nature 540, 60–68 (2016). 27905440 10.1038/nature20566

[12] D. Krueger-Burg, T. Papadopoulos, N. Brose, Organizers of inhibitory synapses come of age. Curr. Opin. Neurobiol. 45, 66–77 (2017). 28460365 10.1016/j.conb.2017.04.003

[13] S. A. Connor, T. J. Siddiqui, Synapse organizers as molecular codes for synaptic plasticity. Trends Neurosci. 46, 971–985 (2023). 37652840 10.1016/j.tins.2023.08.001

[14] F. Koopmans, P. van Nierop, M. Andres-Alonso, A. Byrnes, T. Cijsouw, M. P. Coba, L. N. Cornelisse, R. J. Farrell, H. L. Goldschmidt, D. P. Howrigan, N. K. Hussain, C. Imig, A. P. H. de Jong, H. Jung, M. Kohansalnodehi, B. Kramarz, N. Lipstein, R. C. Lovering, H. MacGillavry, V. Mariano, H. Mi, M. Ninov, D. Osumi-Sutherland, R. Pielot, K. H. Smalla, H. Tang, K. Tashman, R. F. G. Toonen, C. Verpelli, R. Reig-Viader, K. Watanabe, J. van Weering, T. Achsel, G. Ashrafi, N. Asi, T. C. Brown, P. De Camilli, M. Feuermann, R. E. Foulger, P. Gaudet, A. Joglekar, A. Kanellopoulos, R. Malenka, R. A. Nicoll, C. Pulido, J. de Juan-Sanz, M. Sheng, T. C. Sudhof, H. U. Tilgner, C. Bagni, A. Bayes, T. Biederer, N. Brose, J. J. E. Chua, D. C. Dieterich, E. D. Gundelfinger, C. Hoogenraad, R. L. Huganir, R. Jahn, P. S. Kaeser, E. Kim, M. R. Kreutz, P. S. McPherson, B. M. Neale, V. O'Connor, D. Posthuma, T. A. Ryan, C. Sala, G. Feng, S. E. Hyman, P. D. Thomas, A. B. Smit, M. Verhage, SynGO: An evidence-based, expert-curated knowledge base for the synapse. Neuron 103, 217–234.e4 (2019). 31171447 10.1016/j.neuron.2019.05.002 PMC6764089

[15] C. Rosenmund, J. Rettig, N. Brose, Molecular mechanisms of active zone function. Curr. Opin. Neurobiol. 13, 509–519 (2003). 14630212 10.1016/j.conb.2003.09.011

[16] M. Gassmann, B. Bettler, Regulation of neuronal GABA_B_ receptor functions by subunit composition. Nat. Rev. Neurosci. 13, 380–394 (2012). 22595784 10.1038/nrn3249

[17] R. Vigot, S. Barbieri, H. Brauner-Osborne, R. Turecek, R. Shigemoto, Y. P. Zhang, R. Lujan, L. H. Jacobson, B. Biermann, J. M. Fritschy, C. M. Vacher, M. Muller, G. Sansig, N. Guetg, J. F. Cryan, K. Kaupmann, M. Gassmann, T. G. Oertner, B. Bettler, Differential compartmentalization and distinct functions of GABA_B_ receptor variants. Neuron 50, 589–601 (2006). 16701209 10.1016/j.neuron.2006.04.014 PMC3531664

[18] M. L. Cediel, M. Stawarski, X. Blanc, L. Noskova, M. Magner, K. Platzer, J. Gburek-Augustat, D. Baldridge, J. N. Constantino, E. Ranza, B. Bettler, S. E. Antonarakis, GABBR1 monoallelic de novo variants linked to neurodevelopmental delay and epilepsy. Am. J. Hum. Genet. 109, 1885–1893 (2022). 36103875 10.1016/j.ajhg.2022.08.010 PMC9606381

[19] V. Jakob, A. Schreiner, R. Tikkanen, A. Starzinski-Powitz, Targeting of transmembrane protein shrew-1 to adherens junctions is controlled by cytoplasmic sorting motifs. Mol. Biol. Cell 17, 3397–3408 (2006). 16707570 10.1091/mbc.E05-11-1034 PMC1525240

[20] N. Sobreira, F. Schiettecatte, D. Valle, A. Hamosh, GeneMatcher: A matching tool for connecting investigators with an interest in the same gene. Hum. Mutat. 36, 928–930 (2015). 26220891 10.1002/humu.22844 PMC4833888

[21] P. Rentzsch, M. Schubach, J. Shendure, M. Kircher, CADD-splice-improving genome-wide variant effect prediction using deep learning-derived splice scores. Genome Med. 13, 31 (2021). 33618777 10.1186/s13073-021-00835-9 PMC7901104

[22] M. Khajavi, K. Inoue, J. R. Lupski, Nonsense-mediated mRNA decay modulates clinical outcome of genetic disease. Eur. J. Hum. Genet. 14, 1074–1081 (2006). 16757948 10.1038/sj.ejhg.5201649

[23] L. Linde, S. Boelz, G. Neu-Yilik, A. E. Kulozik, B. Kerem, The efficiency of nonsense-mediated mRNA decay is an inherent character and varies among different cells. Eur. J. Hum. Genet. 15, 1156–1162 (2007). 17625509 10.1038/sj.ejhg.5201889

[24] A. B. Zetoune, S. Fontaniere, D. Magnin, O. Anczukow, M. Buisson, C. X. Zhang, S. Mazoyer, Comparison of nonsense-mediated mRNA decay efficiency in various murine tissues. BMC Genet. 9, 83 (2008). 19061508 10.1186/1471-2156-9-83 PMC2607305

[25] H. Sato, R. H. Singer, Cellular variability of nonsense-mediated mRNA decay. Nat. Commun. 12, 7203 (2021). 34893608 10.1038/s41467-021-27423-0 PMC8664836

[26] V. Sereikaite, T. Fritzius, V. B. Kasaragod, N. Bader, H. M. Maric, H. Schindelin, B. Bettler, K. Stromgaard, Targeting the γ-aminobutyric acid type B (GABA_B_) receptor complex: Development of inhibitors targeting the K^+^ channel tetramerization domain (KCTD) containing proteins/GABA_B_ receptor protein-protein interaction. J. Med. Chem. 62, 8819–8830 (2019). 31509708 10.1021/acs.jmedchem.9b01087

[27] H. E. Scharfman, The enigmatic mossy cell of the dentate gyrus. Nat. Rev. Neurosci. 17, 562–575 (2016). 27466143 10.1038/nrn.2016.87 PMC5369357

[28] M. S. Cembrowski, L. Wang, K. Sugino, B. C. Shields, N. Spruston, Hipposeq: A comprehensive RNA-seq database of gene expression in hippocampal principal neurons. eLife 5, e14997 (2016). 27113915 10.7554/eLife.14997 PMC4846374

[29] E. Kordeli, J. Davis, B. Trapp, V. Bennett, An isoform of ankyrin is localized at nodes of Ranvier in myelinated axons of central and peripheral nerves. J. Cell Biol. 110, 1341–1352 (1990). 2139035 10.1083/jcb.110.4.1341 PMC2116078

[30] S. K. Tyagarajan, J. M. Fritschy, Gephyrin: A master regulator of neuronal function? Nat. Rev. Neurosci. 15, 141–156 (2014). 24552784 10.1038/nrn3670

[31] A. C. Horton, M. D. Ehlers, Neuronal polarity and trafficking. Neuron 40, 277–295 (2003). 14556709 10.1016/s0896-6273(03)00629-9

[32] E. H. Feinberg, M. K. Vanhoven, A. Bendesky, G. Wang, R. D. Fetter, K. Shen, C. I. Bargmann, GFP reconstitution across synaptic partners (GRASP) defines cell contacts and synapses in living nervous systems. Neuron 57, 353–363 (2008). 18255029 10.1016/j.neuron.2007.11.030

[33] P. D. Rem, V. Sereikaite, D. Fernandez-Fernandez, S. Reinartz, D. Ulrich, T. Fritzius, L. Trovo, S. Roux, Z. Chen, P. Rondard, J. P. Pin, J. Schwenk, B. Fakler, M. Gassmann, T. R. Barkat, K. Stromgaard, B. Bettler, Soluble amyloid-β precursor peptide does not regulate GABA_B_ receptor activity. eLife 12, e82082 (2023). 36688536 10.7554/eLife.82082 PMC9917443

[34] T. Manabe, D. J. Wyllie, D. J. Perkel, R. A. Nicoll, Modulation of synaptic transmission and long-term potentiation: Effects on paired pulse facilitation and EPSC variance in the CA1 region of the hippocampus. J. Neurophysiol. 70, 1451–1459 (1993). 7904300 10.1152/jn.1993.70.4.1451

[35] D. Debanne, N. C. Guerineau, B. H. Gahwiler, S. M. Thompson, Paired-pulse facilitation and depression at unitary synapses in rat hippocampus: Quantal fluctuation affects subsequent release. J. Physiol. 491, 163–176 (1996). 9011608 10.1113/jphysiol.1996.sp021204 PMC1158767

[36] L. E. Dobrunz, C. F. Stevens, Heterogeneity of release probability, facilitation, and depletion at central synapses. Neuron 18, 995–1008 (1997). 9208866 10.1016/s0896-6273(00)80338-4

[37] T. Sippy, A. Cruz-Martin, A. Jeromin, F. E. Schweizer, Acute changes in short-term plasticity at synapses with elevated levels of neuronal calcium sensor-1. Nat. Neurosci. 6, 1031–1038 (2003). 12947410 10.1038/nn1117 PMC3132582

[38] J. Vienne, B. Bettler, P. Franken, M. Tafti, Differential effects of GABA_B_ receptor subtypes, γ-hydroxybutyric acid, and baclofen on EEG activity and sleep regulation. J. Neurosci. 30, 14194–14204 (2010). 20962240 10.1523/JNEUROSCI.3145-10.2010 PMC6634755

[39] S. Hannan, M. E. Wilkins, T. G. Smart, Sushi domains confer distinct trafficking profiles on GABA_B_ receptors. Proc. Natl. Acad. Sci. U.S.A. 109, 12171–12176 (2012). 22778417 10.1073/pnas.1201660109 PMC3409743

[40] H. A. Dunn, D. N. Patil, Y. Cao, C. Orlandi, K. A. Martemyanov, Synaptic adhesion protein ELFN1 is a selective allosteric modulator of group III metabotropic glutamate receptors in trans. Proc. Natl. Acad. Sci. U.S.A. 115, 5022–5027 (2018). 29686062 10.1073/pnas.1722498115 PMC5948991

[41] H. A. Dunn, S. Zucca, M. Dao, C. Orlandi, K. A. Martemyanov, ELFN2 is a postsynaptic cell adhesion molecule with essential roles in controlling group III mGluRs in the brain and neuropsychiatric behavior. Mol. Psychiatry 24, 1902–1919 (2019). 31485013 10.1038/s41380-019-0512-3 PMC6874751

[42] N. H. Tomioka, H. Yasuda, H. Miyamoto, M. Hatayama, N. Morimura, Y. Matsumoto, T. Suzuki, M. Odagawa, Y. S. Odaka, Y. Iwayama, J. Won Um, J. Ko, Y. Inoue, S. Kaneko, S. Hirose, K. Yamada, T. Yoshikawa, K. Yamakawa, J. Aruga, Elfn1 recruits presynaptic mGluR7 in trans and its loss results in seizures. Nat. Commun. 5, 4501 (2014). 25047565 10.1038/ncomms5501

[43] L. Backwell, J. A. Marsh, Diverse molecular mechanisms underlying pathogenic protein mutations: Beyond the loss-of-function paradigm. Annu. Rev. Genomics Hum. Genet. 23, 475–498 (2022). 35395171 10.1146/annurev-genom-111221-103208

[44] F. Luo, A. Sclip, S. Merrill, T. C. Sudhof, Neurexins regulate presynaptic GABA_B_-receptors at central synapses. Nat. Commun. 12, 2380 (2021). 33888718 10.1038/s41467-021-22753-5 PMC8062527

[45] F. Luo, A. Sclip, M. Jiang, T. C. Sudhof, Neurexins cluster Ca^2+^ channels within the presynaptic active zone. EMBO J. 39, e103208 (2020). 32134527 10.15252/embj.2019103208 PMC7110102

[46] L. Y. Chen, M. Jiang, B. Zhang, O. Gokce, T. C. Sudhof, Conditional deletion of all neurexins defines diversity of essential synaptic organizer functions for neurexins. Neuron 94, 611–625.e4 (2017). 28472659 10.1016/j.neuron.2017.04.011 PMC5501922

[47] C. H. Davies, S. J. Starkey, M. F. Pozza, G. L. Collingridge, GABA autoreceptors regulate the induction of LTP. Nature 349, 609–611 (1991). 1847993 10.1038/349609a0

[48] M. Lek, K. J. Karczewski, E. V. Minikel, K. E. Samocha, E. Banks, T. Fennell, A. H. O'Donnell-Luria, J. S. Ware, A. J. Hill, B. B. Cummings, T. Tukiainen, D. P. Birnbaum, J. A. Kosmicki, L. E. Duncan, K. Estrada, F. Zhao, J. Zou, E. Pierce-Hoffman, J. Berghout, D. N. Cooper, N. Deflaux, M. DePristo, R. Do, J. Flannick, M. Fromer, L. Gauthier, J. Goldstein, N. Gupta, D. Howrigan, A. Kiezun, M. I. Kurki, A. L. Moonshine, P. Natarajan, L. Orozco, G. M. Peloso, R. Poplin, M. A. Rivas, V. Ruano-Rubio, S. A. Rose, D. M. Ruderfer, K. Shakir, P. D. Stenson, C. Stevens, B. P. Thomas, G. Tiao, M. T. Tusie-Luna, B. Weisburd, H. H. Won, D. Yu, D. M. Altshuler, D. Ardissino, M. Boehnke, J. Danesh, S. Donnelly, R. Elosua, J. C. Florez, S. B. Gabriel, G. Getz, S. J. Glatt, C. M. Hultman, S. Kathiresan, M. Laakso, S. McCarroll, M. I. McCarthy, D. McGovern, R. McPherson, B. M. Neale, A. Palotie, S. M. Purcell, D. Saleheen, J. M. Scharf, P. Sklar, P. F. Sullivan, J. Tuomilehto, M. T. Tsuang, H. C. Watkins, J. G. Wilson, M. J. Daly, D. G. MacArthur, Exome Aggregation Consortium, Analysis of protein-coding genetic variation in 60,706 humans. Nature 536, 285–291 (2016). 27535533 10.1038/nature19057 PMC5018207

[49] R. L. Collins, J. T. Glessner, E. Porcu, M. Lepamets, R. Brandon, C. Lauricella, L. Han, T. Morley, L. M. Niestroj, J. Ulirsch, S. Everett, D. P. Howrigan, P. M. Boone, J. Fu, K. J. Karczewski, G. Kellaris, C. Lowther, D. Lucente, K. Mohajeri, M. Noukas, X. Nuttle, K. E. Samocha, M. Trinh, F. Ullah, U. Vosa, Epi25 Consortium, Estonian Biobank Research Team, M. E. Hurles, S. Aradhya, E. E. Davis, H. Finucane, J. F. Gusella, A. Janze, N. Katsanis, L. Matyakhina, B. M. Neale, D. Sanders, S. Warren, J. C. Hodge, D. Lal, D. M. Ruderfer, J. Meck, R. Magi, T. Esko, A. Reymond, Z. Kutalik, H. Hakonarson, S. Sunyaev, H. Brand, M. E. Talkowski, A cross-disorder dosage sensitivity map of the human genome. Cell 185, 3041–3055.e25 (2022). 35917817 10.1016/j.cell.2022.06.036 PMC9742861

[50] H. S. Jin, J. Kim, W. Kwak, H. Jeong, G. B. Lim, C. G. Lee, Identification of a novel mutation in BRD4 that causes autosomal dominant syndromic congenital cataracts associated with other neuro-skeletal anomalies. PLOS ONE 12, e0169226 (2017). 28076398 10.1371/journal.pone.0169226 PMC5226720

[51] G. Jouret, S. Heide, A. Sorlin, L. Faivre, S. Chantot-Bastaraud, C. Beneteau, M. Denis-Musquer, P. D. Turnpenny, C. Coutton, G. Vieville, J. Thevenon, A. Larson, F. Petit, E. Boudry, T. Smol, B. Delobel, B. Duban-Bedu, C. Fallerini, F. Mari, C. Lo Rizzo, A. Renieri, J. H. Caberg, A. S. Denomme-Pichon, F. Tran Mau-Them, I. Maystadt, T. Courtin, B. Keren, L. Mouthon, P. Charles, S. Cuinat, B. Isidor, P. Theis, C. Muller, M. Kulisic, S. Turkmen, D. Stieber, D. Bourgeois, E. Scalais, B. Klink, Understanding the new BRD4-related syndrome: Clinical and genomic delineation with an international cohort study. Clin. Genet. 102, 117–122 (2022). 35470444 10.1111/cge.14141

[52] G. Olley, M. Ansari, H. Bengani, G. R. Grimes, J. Rhodes, A. von Kriegsheim, A. Blatnik, F. J. Stewart, E. Wakeling, N. Carroll, A. Ross, S. M. Park, Deciphering Developmental Disorders Study, W. A. Bickmore, M. M. Pradeepa, D. R. FitzPatrick, BRD4 interacts with NIPBL and BRD4 is mutated in a Cornelia de Lange-like syndrome. Nat. Genet. 50, 329–332 (2018). 29379197 10.1038/s41588-018-0042-y PMC6469577

[53] J. Thevenon, E. Lopez, B. Keren, D. Heron, C. Mignot, C. Altuzarra, M. Beri-Dexheimer, C. Bonnet, E. Magnin, L. Burglen, D. Minot, J. Vigneron, S. Morle, M. Anheim, P. Charles, A. Brice, L. Gallagher, J. Amiel, E. Haffen, C. Mach, C. Depienne, D. Doummar, M. Bonnet, L. Duplomb, V. Carmignac, P. Callier, N. Marle, A. L. Mosca-Boidron, V. Roze, B. Aral, F. Razavi, P. Jonveaux, L. Faivre, C. Thauvin-Robinet, Intragenic CAMTA1 rearrangements cause non-progressive congenital ataxia with or without intellectual disability. J. Med. Genet. 49, 400–408 (2012). 22693284 10.1136/jmedgenet-2012-100856

[54] M. Shinawi, R. Coorg, J. S. Shimony, D. K. Grange, H. Al-Kateb, Intragenic CAMTA1 deletions are associated with a spectrum of neurobehavioral phenotypes. Clin. Genet. 87, 478–482 (2015). 24738973 10.1111/cge.12407

[55] M. Vidal, M. E. Cusick, A. L. Barabasi, Interactome networks and human disease. Cell 144, 986–998 (2011). 21414488 10.1016/j.cell.2011.02.016 PMC3102045

[56] E. L. Huttlin, R. J. Bruckner, J. A. Paulo, J. R. Cannon, L. Ting, K. Baltier, G. Colby, F. Gebreab, M. P. Gygi, H. Parzen, J. Szpyt, S. Tam, G. Zarraga, L. Pontano-Vaites, S. Swarup, A. E. White, D. K. Schweppe, R. Rad, B. K. Erickson, R. A. Obar, K. G. Guruharsha, K. Li, S. A. Rtavanis-Tsakonas, S. P. Gygi, J. W. Harper, Architecture of the human interactome defines protein communities and disease networks. Nature 545, 505–509 (2017). 28514442 10.1038/nature22366 PMC5531611

[57] A. Pagano, G. Rovelli, J. Mosbacher, T. Lohmann, B. Duthey, D. Stauffer, D. Ristig, V. Schuler, I. Meigel, C. Lampert, T. Stein, L. Prezeau, J. Blahos, J. Pin, W. Froestl, R. Kuhn, J. Heid, K. Kaupmann, B. Bettler, C-terminal interaction is essential for surface trafficking but not for heteromeric assembly of GABA_B_ receptors. J. Neurosci. 21, 1189–1202 (2001). 11160389 10.1523/JNEUROSCI.21-04-01189.2001 PMC6762227

[58] L. Adelfinger, R. Turecek, K. Ivankova, A. A. Jensen, S. J. Moss, M. Gassmann, B. Bettler, GABA_B_ receptor phosphorylation regulates KCTD12-induced K^+^ current desensitization. Biochem. Pharmacol. 91, 369–379 (2014). 25065880 10.1016/j.bcp.2014.07.013 PMC4402209

[59] O. M. Subach, I. S. Gundorov, M. Yoshimura, F. V. Subach, J. Zhang, D. Gruenwald, E. A. Souslova, D. M. Chudakov, V. V. Verkhusha, Conversion of red fluorescent protein into a bright blue probe. Chem. Biol. 15, 1116–1124 (2008). 18940671 10.1016/j.chembiol.2008.08.006 PMC2585067

[60] Z. Nikolovska-Coleska, R. Wang, X. Fang, H. Pan, Y. Tomita, P. Li, P. P. Roller, K. Krajewski, N. G. Saito, J. A. Stuckey, S. Wang, Development and optimization of a binding assay for the XIAP BIR3 domain using fluorescence polarization. Anal. Biochem. 332, 261–273 (2004). 15325294 10.1016/j.ab.2004.05.055

[61] C. A. Sailer, H. Hu, W. A. Kaufmann, M. Trieb, C. Schwarzer, J. F. Storm, H. G. Knaus, Regional differences in distribution and functional expression of small-conductance Ca^2+^-activated K^+^ channels in rat brain. J. Neurosci. 22, 9698–9707 (2002). 12427825 10.1523/JNEUROSCI.22-22-09698.2002 PMC6757844

[62] M. K. Kocylowski, H. Aypek, W. Bildl, M. Helmstadter, P. Trachte, B. Dumoulin, S. Wittosch, L. Kuhne, U. Aukschun, C. Teetzen, O. Kretz, B. Gaal, A. Kulik, C. Antignac, G. Mollet, A. Kottgen, B. Gocmen, J. Schwenk, U. Schulte, T. B. Huber, B. Fakler, F. Grahammer, A slit-diaphragm-associated protein network for dynamic control of renal filtration. Nat. Commun. 13, 6446 (2022). 36307401 10.1038/s41467-022-33748-1 PMC9616960

[63] J. Y. Tiao, A. Bradaia, B. Biermann, K. Kaupmann, M. Metz, C. Haller, A. G. Rolink, E. Pless, P. N. Barlow, M. Gassmann, B. Bettler, The sushi domains of secreted GABA_B1_ isoforms selectively impair GABA_B_ heteroreceptor function. J. Biol. Chem. 283, 31005–31011 (2008). 18765663 10.1074/jbc.M804464200 PMC2576543

[64] P. Bhandari, D. Vandael, D. Fernandez-Fernandez, T. Fritzius, D. Kleindienst, C. Onal, J. Montanaro, M. Gassmann, P. Jonas, A. Kulik, B. Bettler, R. Shigemoto, P. Koppensteiner, GABA_B_ receptor auxiliary subunits modulate Cav2.3-mediated release from medial habenula terminals. eLife 10, e68274 (2021). 33913808 10.7554/eLife.68274 PMC8121548

[65] A. Cardona, S. Saalfeld, J. Schindelin, I. Arganda-Carreras, S. Preibisch, M. Longair, P. Tomancak, V. Hartenstein, R. J. Douglas, TrakEM2 software for neural circuit reconstruction. PLOS ONE 7, e38011 (2012). 22723842 10.1371/journal.pone.0038011 PMC3378562

[66] A. Matsubara, J. H. Laake, S. Davanger, S. Usami, O. P. Ottersen, Organization of AMPA receptor subunits at a glutamate synapse: A quantitative immunogold analysis of hair cell synapses in the rat organ of Corti. J. Neurosci. 16, 4457–4467 (1996). 8699256 10.1523/JNEUROSCI.16-14-04457.1996 PMC6578857

[67] S. Boudkkazi, A. Brechet, J. Schwenk, B. Fakler, Cornichon2 dictates the time course of excitatory transmission at individual hippocampal synapses. Neuron 82, 848–858 (2014). 24853943 10.1016/j.neuron.2014.03.031

[68] A. Vassalli, P. Franken, Hypocretin (orexin) is critical in sustaining theta/gamma-rich waking behaviors that drive sleep need. Proc. Natl. Acad. Sci. U.S.A. 114, E5464–E5473 (2017). 28630298 10.1073/pnas.1700983114 PMC5502606

