# Supplementary material for: Monoallelic de novo AJAP1 loss-of-function variants disrupt trans-synaptic control of neurotransmitter release
Source: Sci Adv. 2024 Jul 10;10(28):eadk5462. doi: 10.1126/sciadv.adk5462 (PMC11235169; doi:10.1126/sciadv.adk5462)
Supplement: Supplementary file 1 — Figs. S1 to S9 Legend for data S1 Legends for movies S1 to S3 [file sciadv.adk5462_sm.pdf]

Science Advances
AAAS

Supplementary Materials for

Monoallelic de novo *AJAP1* loss-of-function variants disrupt trans-synaptic control of neurotransmitter release

Simon Früh *et al.*

Corresponding author: Bernhard Bettler, bernhard.bettler@unibas.ch

Sci. Adv. **10**, eadk5462 (2024)
DOI: 10.1126/sciadv.adk5462

The PDF file includes:

Figs. S1 to S9
Legend for data S1
Legends for movies S1 to S3

Other Supplementary Material for this manuscript includes the following:

Data S1
Movies S1 to S3

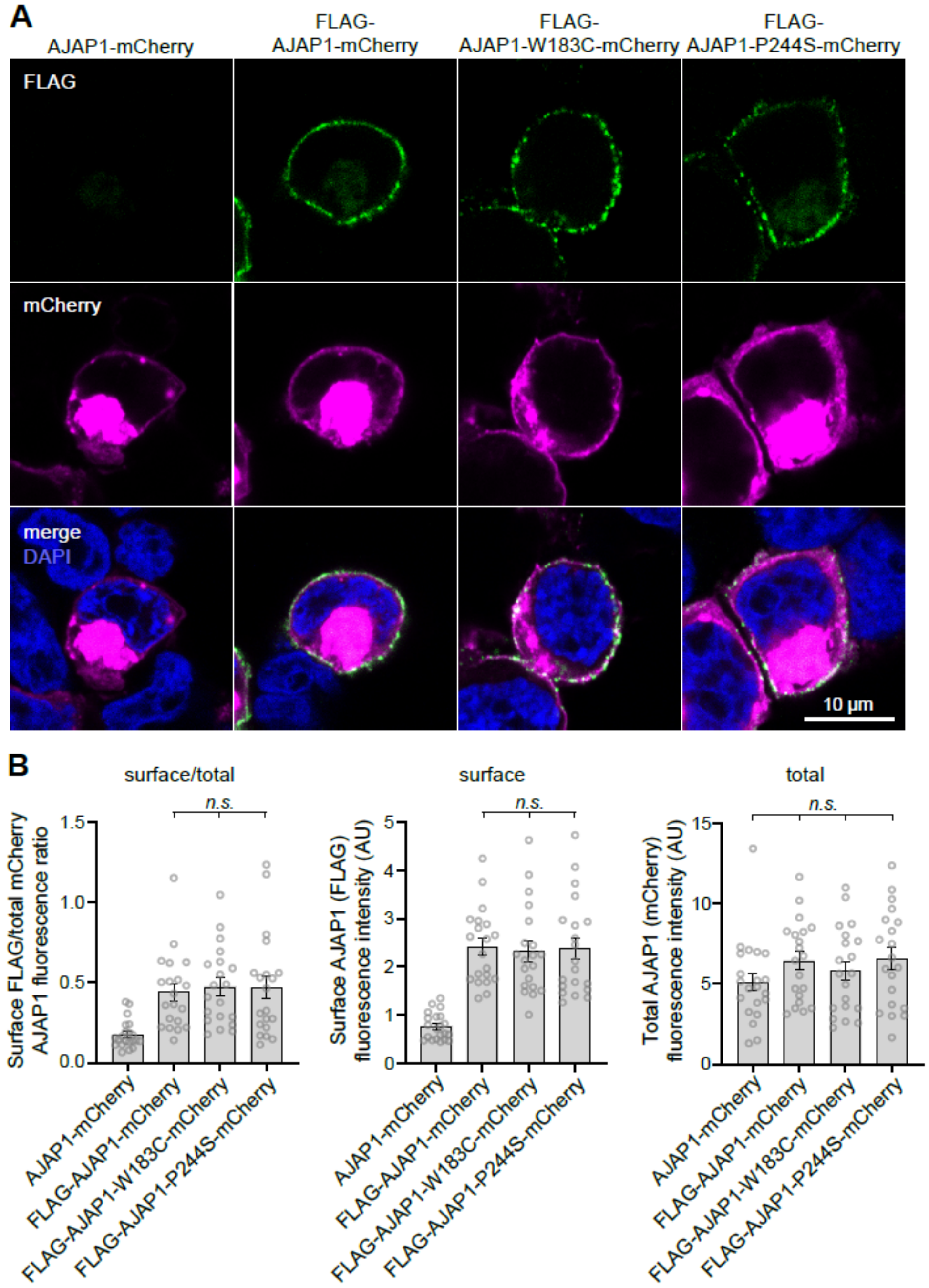

Fig. S1. Cell surface expression of the AJAP-W183C and AJAP1-P244S variants.
(**A**) AJAP1 constructs N- and C-terminally tagged with FLAG and mCherry, respectively, expressed in HEK293T cells. Cell surface AJAP1 expression was determined in non-permeabilized cells using anti-Flag antibodies and fluorescence labeled secondary antibodies. Total AJAP1 expression was determined by measuring mCherry fluorescence intensity. Expression of AJAP1-mCherry served as a control for anti-FLAG antibody specificity. (**B**) Bar graphs of surface, total and surface-to-total expression ratio of AJAP1. n.s.: $P > 0.05$, Kruskal-Wallis test and Dunn's multiple comparisons test (surface/total and total), one-way ANOVA and Tukey's multiple comparisons test (surface), n=20-21 cells per condition.

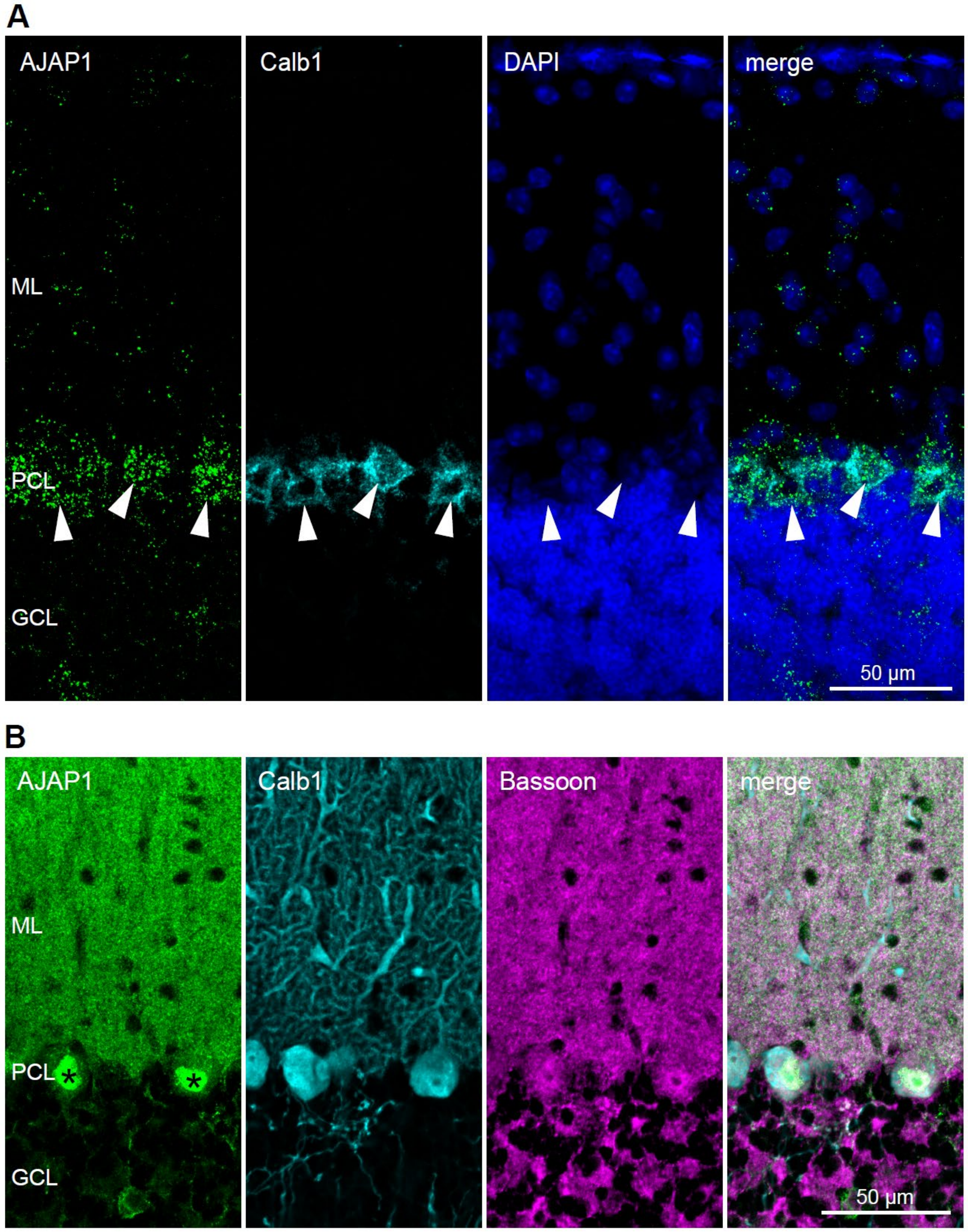

Fig. S2. Cellular and subcellular AJAP1 expression in the cerebellum.
(**A**) FISH using AJAP1 and Calbindin-1 (Calb1) probes in midsagittal cerebellum sections. *Ajap1* and *Calb1* transcripts co-localize in Purkinje cells (arrowheads). Calb1 and DAPI were used as a marker of Purkinje cells and to stain nuclei, respectively. (**B**) AJAP1 and Calb1 immunolabeling in midsagittal cerebellum sections. The molecular layer exhibits strongest AJAP1 labeling, consistent with AJAP1 localization in Purkinje cell dendrites. Asterisks denote unspecific AJAP1 immunolabeling of Purkinje cell somata (see Fig. S6 for *Ajap1*$^{-/-}$ control). Bassoon was used as a presynaptic marker. ML, molecular layer; PCL, Purkinje cell layer; GCL, granule cell layer.

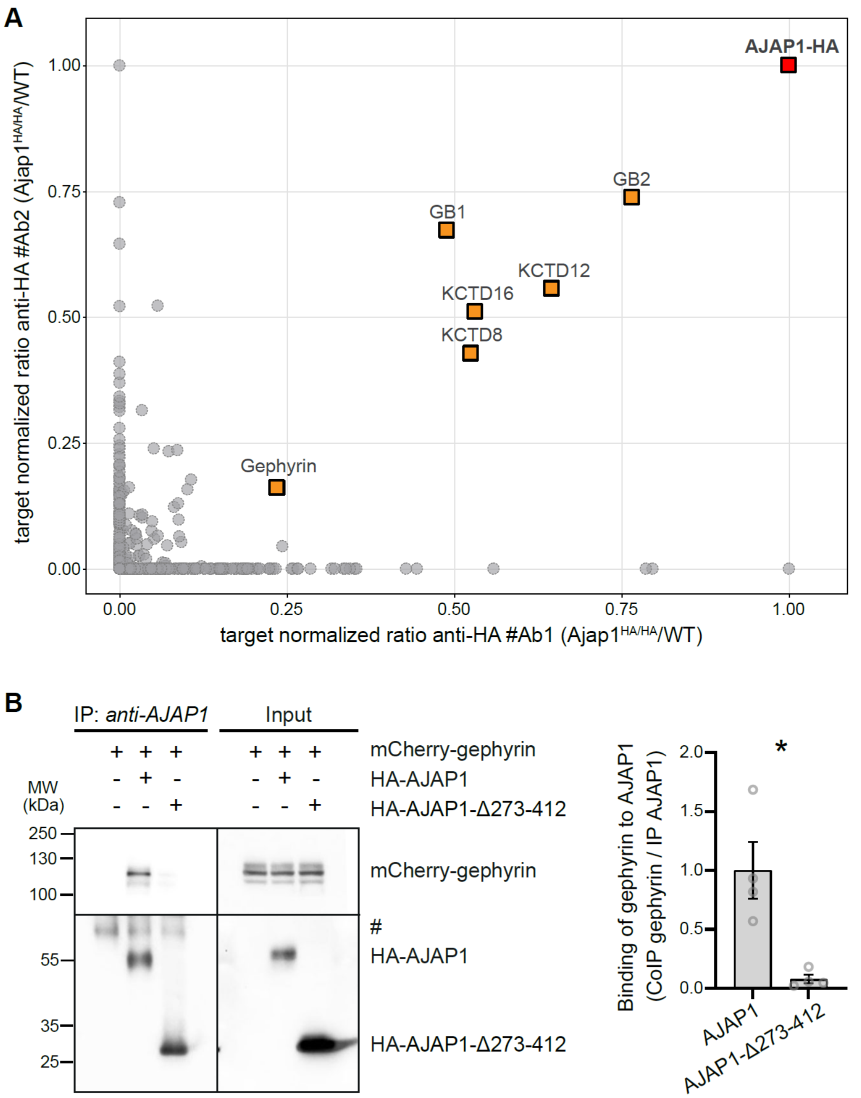

Fig. S3. Proteomic identification of AJAP1-interacting proteins in solubilized mouse brain membranes.

(**A**) Abundance ratios of all proteins identified in affinity purifications from solubilized *Ajap1*$^{HA/HA}$ and control WT mouse brain membranes using two anti-HA antibodies (target-normalized ratio values *Ajap1*$^{HA/HA}$ versus WT, see Methods). Ratio plots show that GB1, GB2, KCTD proteins and gephyrin (in small quantities) specifically and consistently co-purify in anti-HA affinity purifications from *Ajap1*$^{HA/HA}$ brain membranes. (**B**) Gephyrin binds to the intracellular domain of AJAP1. Representative immunoblot of immunoprecipitation experiments with lysates of transfected HEK293T cells (left) and quantification of co-immunoprecipitation experiments (right). mCherry-tagged gephyrin was expressed along with either HA-tagged AJAP1 or C-terminally truncated AJAP1-Δ273-412. AJAP1 constructs were immunoprecipitated using a polyclonal antibody against the extracellular domain of AJAP1. Input lysates and immunoprecipitations (IP) were analyzed on immunoblots using antibodies recognizing gephyrin and AJAP1. # indicates the heavy chain of the anti-AJAP1 antibody used for immunoprecipitation. For quantification, the gephyrin signal was normalized to the AJAP1 and AJAP1-Δ273-412 signal, respectively, and expressed as a fraction of the AJAP1 condition. *$P < 0.05$, Welch's t-test, n=4 independent experiments.

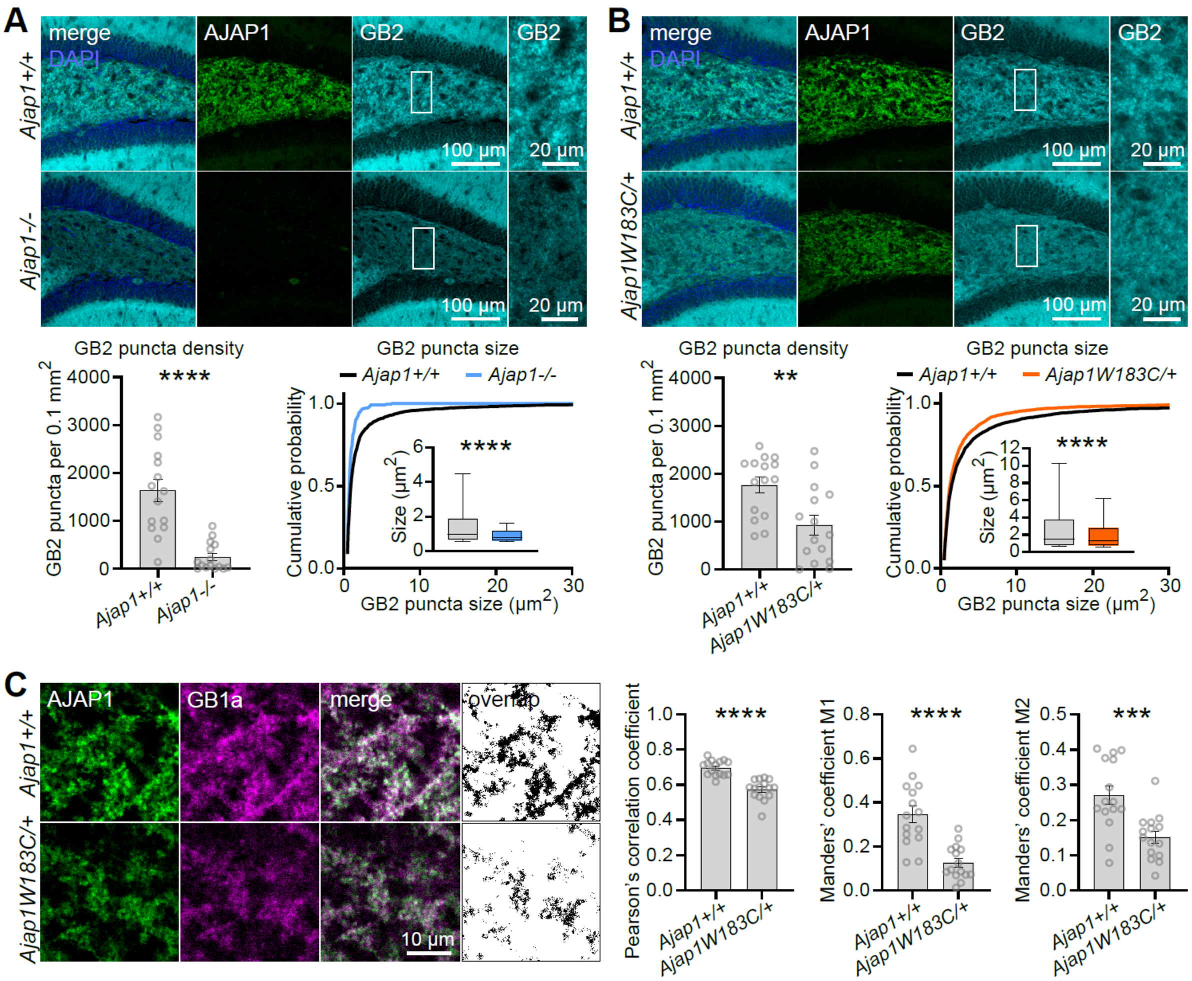

Fig. S4. Reduced GBR protein expression and diminished co-localization of GB1a with AJAP1 protein in the hilus of *Ajap1*$^{-/-}$ and *Ajap1*$^{\mathrm{W183C/+}}$ mice.

(**A**) AJAP1 and GB2 immunofluorescence labeling in the hilus of the dentate gyrus of *Ajap1*$^{-/-}$ and *Ajap1*$^{+/+}$ littermate mice. Density and size of GB2 puncta in the neuropil was significantly reduced in *Ajap1*$^{-/-}$ mice. ****$P < 0.0001$, unpaired t-test with Welch's correction (density), Kolmogorov-Smirnov test (size), n=15 sections from 5 mice per genotype (density), n=1763 puncta from 5 *Ajap1*$^{+/+}$ mice and n=260 puncta from 5 *Ajap1*$^{-/-}$ mice for (size). (**B**) Immunofluorescence labeling of AJAP1 and GB2 in *Ajap1*$^{\mathrm{W183C/+}}$ and *Ajap1*$^{+/+}$ littermate mice. Density and size of GB2 puncta in the neuropil was significantly reduced in *Ajap1*$^{\mathrm{W183C/+}}$ mice. **$P < 0.01$, ****$P < 0.0001$, unpaired t-test (density), Kolmogorov-Smirnov test (size), n=15 sections from 5 mice per genotype (density), n=3231 puncta from 5 *Ajap1*$^{+/+}$ mice and n=1703 puncta from 5 *Ajap1*$^{\mathrm{W183C/+}}$ mice (size). In box plots, boxes represent 25th to 75th percentiles, whiskers represent 10th to 90th percentiles, and the line represents the median. (**C**) Co-localization analysis of AJAP1 and GB1a immunofluorescence labeling in the hilus of *Ajap1*$^{+/+}$ *and Ajap1*$^{\mathrm{W183C/+}}$ mice. Merged and overlap images reveal co-localization of the AJAP1 and GB1a immunofluorescence. Pearson's correlation coefficient and Manders' M1 (fraction of AJAP1 overlapping with GB1a) and Manders' M2 (fraction of GB1a overlapping with AJAP1) co-localization coefficients were significantly reduced with *Ajap1*$^{\mathrm{W183C/+}}$ mice. ****$P < 0.0001$, ***$P < 0.001$, Mann-Whitney test (Pearson's correlation coefficient), unpaired t-test (Manders' co-localization coefficients), n=15 sections from 5 mice per genotype.

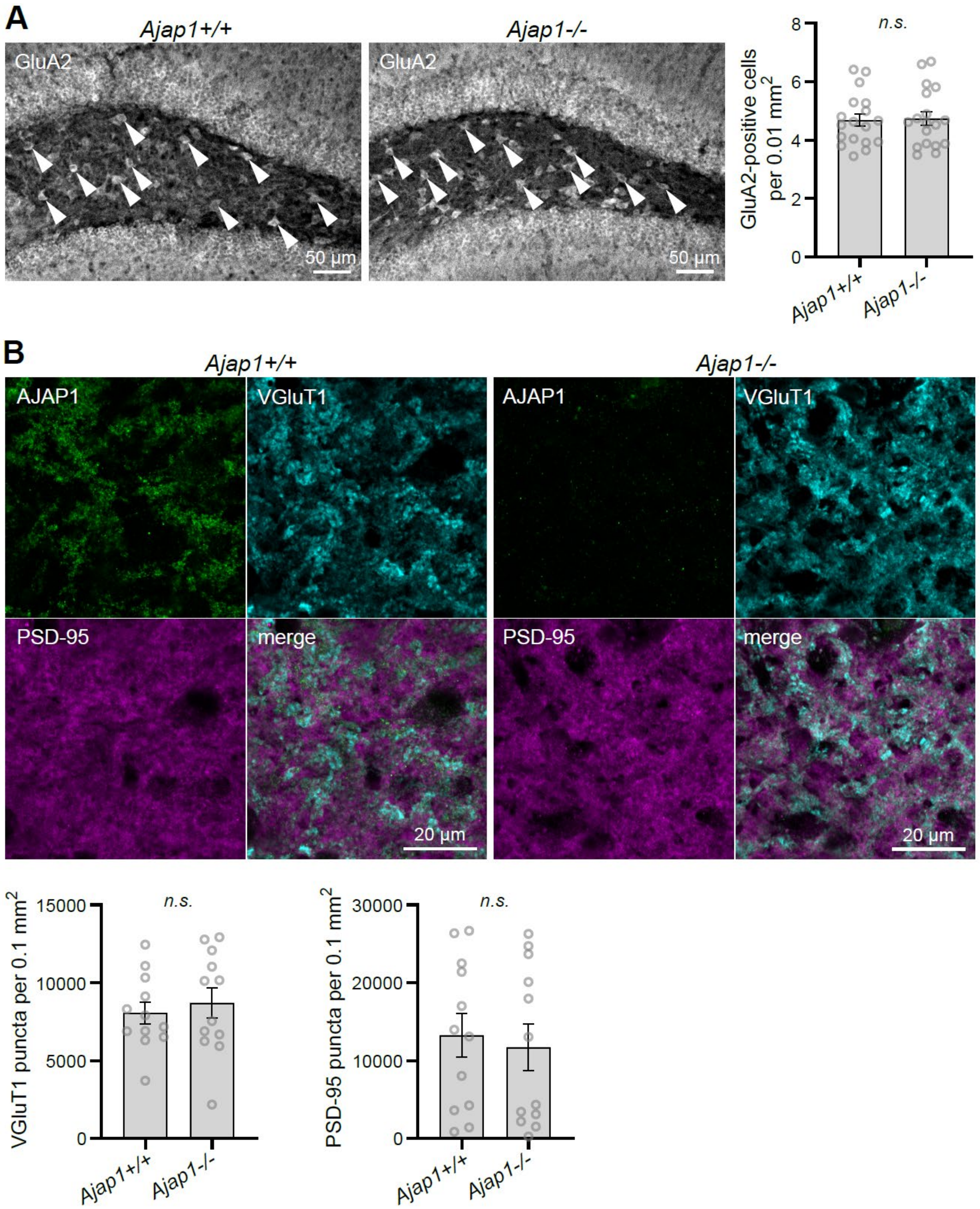

Fig. S5. Immunohistochemical characterization of *Ajap1*$^{-/-}$ mice in the hilus of the dentate gyrus.

(**A**) Representative images of GluA2 immunostaining, a marker for HMCs (arrowheads), in *Ajap1*$^{-/-}$ and *Ajap1*$^{+/+}$ littermate mice. Bar graph shows no difference in the density of HMCs between genotypes. n.s.: $P > 0.05$, unpaired t-test, n=18 sections from 6 mice per genotype. (**B**) Representative images of VGluT1 and PSD-95 immunolabeling in the hilus of the dentate gyrus of *Ajap1*$^{-/-}$ and *Ajap1*$^{+/+}$ littermate mice. Bar graphs show no difference in the number of VGluT1 and PSD-95 puncta between genotypes. n.s.: $P > 0.05$, unpaired t-test (VGluT1), Mann-Whitney test (PSD-95), n=12 sections from 4 mice per genotype.

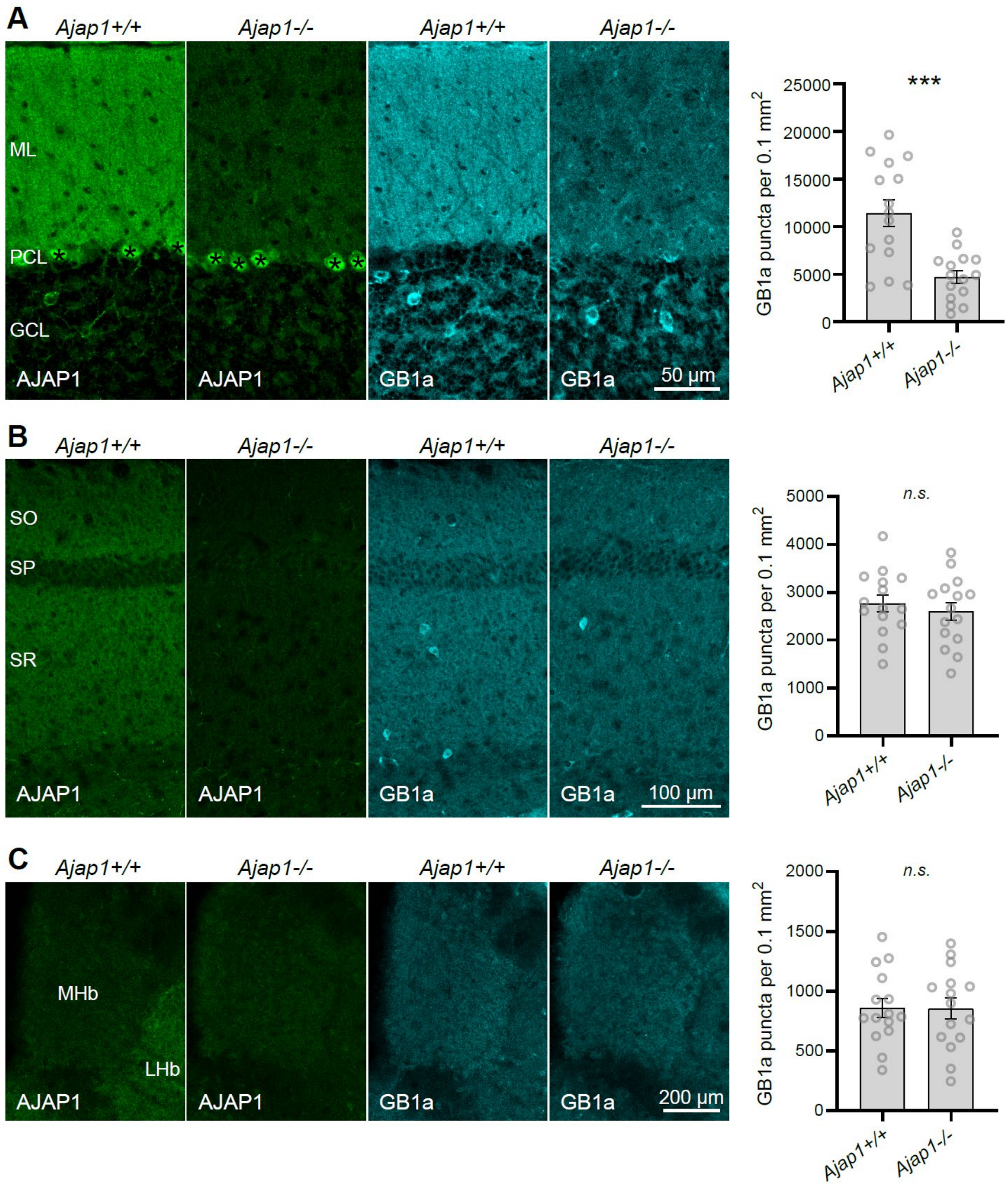

Fig. S6. GB1a protein levels in the cerebellum, CA1 area of the hippocampus, and the medial habenula of *Ajap1*$^{-/-}$ mice.

(**A**) AJAP1 and GB1a immunofluorescence labeling in midsagittal cerebellum sections of *Ajap1*$^{-/-}$ and *Ajap1*$^{+/+}$ littermate mice. Asterisks denote unspecific AJAP1 immunolabeling of Purkinje cell somata. ML, molecular layer; PCL, Purkinje cell layer; GCL, granule cell layer. The density of GB1a puncta in the neuropil of the molecular layer is significantly reduced in *Ajap1*$^{-/-}$ mice. ***$P < 0.001$, unpaired t-test with Welch's correction, n=15 sections from 5 mice per genotype. (**B**) AJAP1 and GB1a immunofluorescence labeling in the CA1 area of the hippocampus in *Ajap1*$^{-/-}$ and *Ajap1*$^{+/+}$ mice. Bar graphs show GB1a puncta density in CA1 stratum radiatum. SO, stratum oriens; SP, stratum pyramidale; SR, stratum radiatum. n.s.: $P > 0.05$, unpaired t-test. (**C**) AJAP1 and GB1a immunofluorescence labeling in the medial habenula of *Ajap1*$^{-/-}$ and *Ajap1*$^{+/+}$ mice. Bar graphs show GB1a puncta density in the medial habenula. MHb, medial habenula; LHb, lateral habenula. n.s.: $P > 0.05$, unpaired t-test.

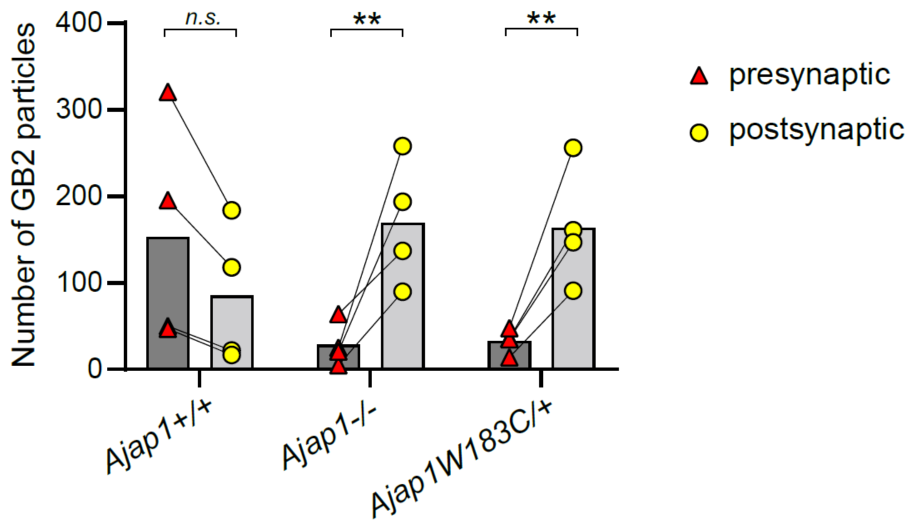

Fig. S7. Pre- and postsynaptic GB2 particle numbers from quantitative electron microscopic analysis.

Pre- and postsynaptic GB2 particles per analyzed segment of dentate gyrus hilus in *Ajap1*$^{+/+}$, *Ajap1*$^{-/-}$, and *Ajap1*$^{W183C/+}$ mice. There are significantly fewer presynaptic than postsynaptic GB2 particles in *Ajap1*$^{-/-}$ and *Ajap1*$^{W183C/+}$ mice. In *Ajap1*$^{+/+}$ mice, pre- and postsynaptic GB2 particle numbers are not significantly different. n.s.: $P > 0.05$, $^{**}P < 0.01$, two-way ANOVA and Sidak's multiple comparisons test, n=4 segments from 2 mice per genotype.

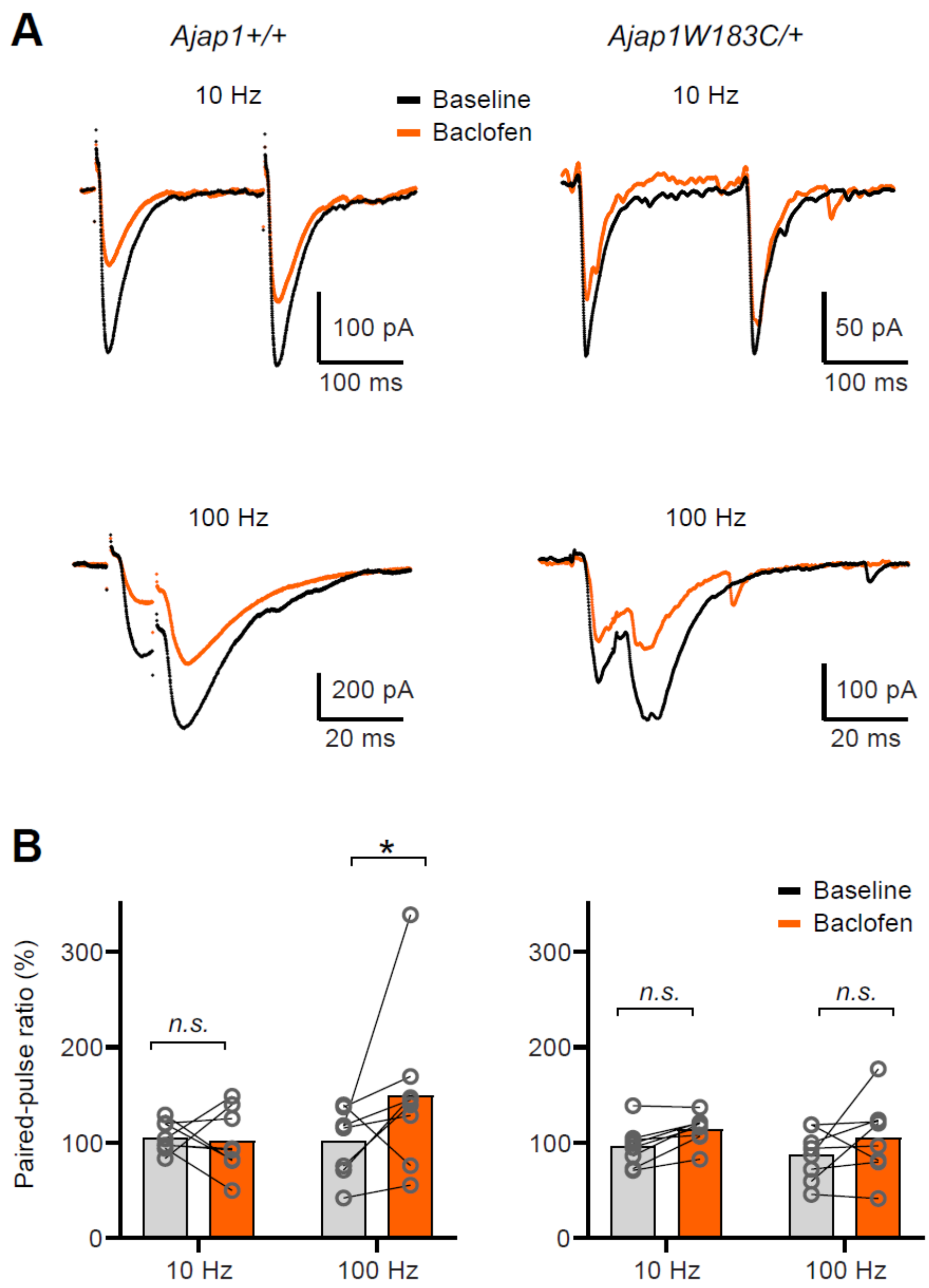

Fig. S8. Influence of baclofen on the PPR of EPSCs at HMC synapses of *Ajap1*$^{+/+}$ and *Ajap1*$^{W183C/+}$ mice.

(**A**) Traces of evoked EPSCs recorded in *Ajap1*$^{+/+}$ (left) and *Ajap1*$^{W183C/+}$ HMCs (right) by paired-pulse stimulation of the CA3 stratum lucidum. Interstimulus intervals were 100 ms (top, 10Hz) and 10 ms (bottom, 100Hz). EPSCs were recorded in the absence (black) and presence (red) of baclofen. (**B**) Bar graphs of the PPRs determined in experiments as in (**A**). PPR values represent the ratio of the second EPSC amplitude to that of the first EPSC amplitude. n.s.: $P > 0.05$, $*P < 0.05$, Wilcoxon matched-pairs test, n=8 cells from 3 mice per genotype.

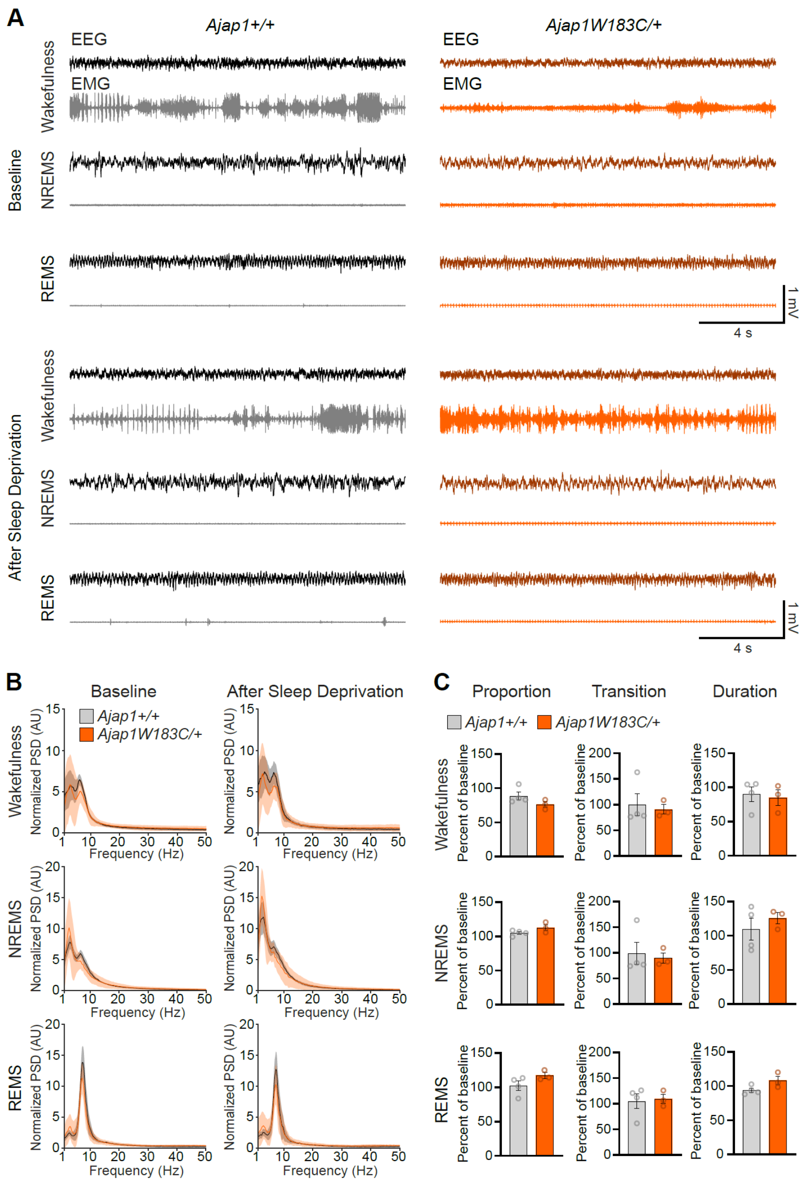

Fig. S9. EEG and EMG recordings of *Ajap1*$^{-/-}$ and *Ajap1*$^{W183C/+}$ mice.

(**A**) Examples of *in vivo* parietal electroencephalography (EEG) and electromyography (EMG) recordings of *Ajap1*$^{+/+}$ and *Ajap1*$^{W183C/+}$ mice before and after 6 h sleep deprivation protocol to detect seizure presence. No seizure was detected throughout 4 days of continuous video-EEG/EMG recording in all mice recorded (n=4 *Ajap1*$^{+/+}$ mice; n=3 *Ajap1*$^{W183C/+}$ mice). For visualization, EEG and EMG signals were high-pass filtered from 0.7 or 10.0 Hz, respectively. (**B**) EEG power spectral density (PSD) profiles for *Ajap1*$^{+/+}$ and *Ajap1*$^{W183C/+}$ mice before and after sleep deprivation for all vigilance states. Lines represent average EEG PSD profiles and shaded regions represent 95% confidence intervals, to which a 20-point moving average filter was applied. (**C**) Vigilance state architecture after sleep deprivation was comparable between *Ajap1*$^{+/+}$ and *Ajap1*$^{W183C/+}$. Vigilance state content was assessed by determining the proportion of time between zeitgeber time 6 to 10 that mice spent in different vigilance states, compared to baseline (before sleep deprivation). Vigilance state stability was assayed by the number of transitions to different vigilance states as well as their average episode durations. n.s.: $P > 0.05$, unpaired t-test.

Data S1 (separate file). Source data for Figure S3A.
Target normalized ratio data for anti-HA #Ab1 and anti-HA #Ab2 presented in Figure S3A.

Movie S1 (separate file). Animation of serial section reconstruction of *Ajap1*$^{+/+}$ dendrite.
Animation of serial section reconstructions of *Ajap1*$^{+/+}$ HMC dendrites. Red dots represent presynaptic and yellow dots represent postsynaptic GB2 particles. Pink areas represent active zones. Related to Figure 6.

Movie S2 (separate file). Animation of serial section reconstruction of *Ajap1*$^{-/-}$ dendrite.
Animation of serial section reconstructions of *Ajap1*$^{-/-}$ HMC dendrites. Red dots represent presynaptic and yellow dots represent postsynaptic GB2 particles. Pink areas represent active zones. Related to Figure 6.

Movie S3 (separate file). Animation of serial section reconstruction of *Ajap1*$^{W183C/+}$ dendrite.
Animation of serial section reconstructions of *Ajap1*$^{W183C/+}$ HMC dendrites. Red dots represent presynaptic and yellow dots represent postsynaptic GB2 particles. Pink areas represent active zones. Related to Figure 6.
